# Atlas of tissue- and developmental stage specific gene expression for the bovine insulin-like growth factor (IGF) system

**DOI:** 10.1371/journal.pone.0200466

**Published:** 2018-07-12

**Authors:** Mani Ghanipoor-Samami, Ali Javadmanesh, Brian M. Burns, Dana A. Thomsen, Greg S. Nattrass, Consuelo Amor S. Estrella, Karen L. Kind, Stefan Hiendleder

**Affiliations:** 1 Robinson Research Institute, The University of Adelaide, Adelaide, South Australia, Australia; 2 JS Davies Epigenetics and Genetics Group, Davies Research Centre, School of Animal and Veterinary Sciences, Roseworthy Campus, The University of Adelaide, Roseworthy, South Australia, Australia; 3 Centre for Animal Science, Queensland Alliance for Agriculture and Food Innovation, The University of Queensland, Rockhampton, Queensland, Australia; 4 Livestock Systems, South Australian Research and Development Institute (SARDI), Roseworthy, South Australia, Australia; Institute of Molecular Genetics of Montpellier, FRANCE

## Abstract

The insulin-like growth factor (IGF) axis is fundamental for mammalian growth and development. However, no comprehensive reference data on gene expression across tissues and pre- and postnatal developmental stages are available for any given species. Here we provide systematic promoter- and splice variant specific information on expression of IGF system components in embryonic (Day 48), fetal (Day 153), term (Day 277, placenta) and juvenile (Day 365–396) tissues of domestic cow, a major agricultural species and biomedical model. Analysis of spatiotemporal changes in expression of IGF1, IGF2, IGF1R, IGF2R, IGFBP1-8 and IR genes, as well as lncRNAs H19 and AIRN, by qPCR, indicated an overall increase in expression from embryo to fetal stage, and decrease in expression from fetal to juvenile stage. The stronger decrease in expression of lncRNAs (average ―16-fold) and ligands (average ―12.1-fold) compared to receptors (average ―5.7-fold) and binding proteins (average ―4.3-fold) is consistent with known functions of IGF peptides and supports important roles of lncRNAs in prenatal development. Pronounced overall reduction in postnatal expression of IGF system components in lung (―12.9-fold) and kidney (―13.2-fold) are signatures of major changes in organ function while more similar hepatic expression levels (―2.2-fold) are evidence of the endocrine rather than autocrine/paracrine role of IGFs in postnatal growth regulation. Despite its rapid growth, placenta displayed a more stable expression pattern than other organs during prenatal development. Quantitative analyses of contributions of promoters P0-P4 to global *IGF2* transcript in fetal tissues revealed that P4 accounted for the bulk of transcript in all tissues but skeletal muscle. Demonstration of *IGF2* expression in fetal muscle and postnatal liver from a promoter orthologous to mouse and human promoter P0 provides further evidence for an evolutionary and developmental shift from placenta-specific P0-expression in rodents and suggests that some aspects of bovine IGF expression may be closer to human than mouse.

## Introduction

The insulin-like growth factor (IGF) system is essential for pre- and postnatal growth and development [1–4] and consists of two growth factors (IGF1, IGF2), type 1 and 2 receptors (IGF1R, IGF2R), the insulin receptor (IR) with short and long isoforms (IR-A, IR-B), six major IGF binding proteins (IGFBP1–6) and several lower-affinity binding proteins (IGFBP7 to IGFBP10) [1, 5, 6]. The IGF1 and IGF2 peptides have strong growth promoting endocrine and paracrine/autocrine actions in a wide range of pre- and postnatal tissues and undergo pronounced changes in expression during prenatal development and after birth [7–13]. Consistently, the IGF1 and IGF2 genes have been identified as quantitative trait loci for growth and development in several mammalian species, including mouse, pig, bovine and human [14–25].

Expression of *IGF1* starts early with transcripts detected in preimplantation stage bovine and human embryos and in midgestation rat embryos [26–28]. Transcription of *IGF1* can be initiated from exon 1 or 2, producing *IGF1* class 1 and 2 mRNAs that yield identical mature IGF1 proteins [29–32]. The IGF2 gene is subject to genomic imprinting and paternally expressed in prenatal mammalian tissues, but switches to biallelic expression in a promoter- and tissue specific manner postnatally [33–38]. Interestingly, in mouse, a sequence in *Igf2* intron 2 encodes for an imprinted miRNA that targets non-imprinted *Igf1* transcripts [39–41]. In sheep, pig, bovine and human, *IGF2* transcripts are expressed from four promoters (*IGF2*-P1-4) in a tissue- and developmental stage specific manner [16, 42–47]. The structure of mouse *Igf2* differs significantly from other mammals and transcripts originate from *Igf2*-P1-P3, which are orthologous to *IGF2*-P2-P4 in species discussed above [48], and an additional placenta-specific promoter (P0). Transcripts equivalent to mouse P0 transcripts have also been identified in human fetal skeletal muscle and several postnatal tissues, including heart, lung, liver, muscle and kidney [49, 50]. Furthermore, in mouse, a previously unknown promoter (Pm) is activated preferentially in mesoderm derived tissues by the expression of antisense *H19* long non-coding RNA (*91H*). This *91H*-mediated *Igf2* activation is counteracted by a large excess of *H19* transcripts [51].

The reciprocally imprinted and maternally expressed long non-coding RNA *H19* is located immediately downstream of *IGF2* and expression of both genes is intrinsically linked through shared control elements such as CTCF binding sites [45, 52–56]. More recent analyses in mouse have shown that *H19* harbors miRNAs, one of which regulates cell proliferation and placental size, most likely by targeting *IGF1R* transcript [57]. Furthermore, correlations between *H19* transcript abundance and bovine fetal skeletal muscle and bone mass suggest that development of other organs may be regulated by *H19* [58, 59].

Both IGF ligands signal through combinations of IGF1R and IR homo- and heterodimers, albeit with different affinities. In bovine and human, alternative splicing of the *IR* transcript produces the two receptor isoforms, IR-A and IR-B, that exclude or include exon 11 [60]. Both form heterodimers with each other and IGF1R [61, 62]. IR-A isoform displays higher affinity for IGF2 than IGF1, while IR-B has a high specificity for insulin [63]. The IGF1 peptide signals through homodimers of IGF1R and heterodimers of IGF1R and IR-A or IR-B, while IGF2 peptide signals through homodimers of IGF1R and IR-A and heterodimers of IGF1R and IR-A or IRA and IR-B [61, 63–65]. The IGF1R gene is expressed ubiquitously and has a major role in maintenance of tissue growth and development [66, 67]. Mutation or ablation of *IGF1R* leads to growth retardation and/or growth failure more severe than *IGF1* deletion [68]. In human, but not mouse, lack of functional *IR* leads to severe intrauterine growth retardation [69, 70].

In contrast to IGF1R and IR receptors, multifunctional IGF2R is primarily a regulator of IGF2 bioavailability and acts as a scavenger receptor that internalizes IGF2 and targets it for lysosomal degradation [71]. However, studies on stimulation of human trophoblast cell invasion by IGF2 suggest intrinsic signaling functions for IGF2R in placenta via the MAPK pathway [72]. The IGF2R gene is imprinted and maternally expressed in all investigated species, including bovine, with the exception of human, where imprinting appears to be polymorphic [73–77]. Ablation of *IGF2R* in mouse results in severe fetal overgrowth [78] and association of *IGF2R* alleles with postnatal growth parameters in cow suggests a general role for IGF2R in growth regulation [79]. Imprinted expression of mouse *Igf2r* is controlled by a reciprocally imprinted antisense of *Igf2r* non-protein coding RNA Airn; orthologues of *Airn* are also expressed in bovine and human [80–82]. However, data on tissue specific developmentally regulated expression of this RNA is lacking.

The IGFBPs modulate bioavailability of IGFs with affinities up to 50-fold higher than IGF1R [83]. Deletion and overexpression models demonstrated organ-specific and general effects of IGFBPs on growth and development [84–87]. The discovery of low affinity IGFBP-related proteins, including IGFBP7 and IGFBP8, has led to the proposal of an IGFBP superfamily [5, 83, 88]. Mice deficient in IGFBP8 die in the perinatal period due to respiratory failure and displayed generalized chondrodysplasia [89].

Expression patterns of genes in the IGF system are highly developmentally regulated [13, 90–100], but changes in tissue specific expression across pre- and postnatal stages by quantitative PCR have not been systematically examined in any species. Here we comprehensively characterize changes in transcript abundances of IGF system genes and associated regulatory long non-coding RNAs in a range of tissues at key developmental time points, i.e., (i) transition from embryo to fetal stage, (ii) fetal stage entering accelerated growth phase and (iii) juvenile stage around puberty. The resulting atlas of tissue- and developmental stage specific expression of the insulin-like growth factor system in bovine is a valuable resource that provides important reference data for future studies of the mammalian IGF system and yields novel insights into similarities and differences between animal model and human.

## Materials and methods

### Animals and tissues

All animal experiments and procedures described in this study were approved by the University of Adelaide, Adelaide, Australia, Animal Ethics Committee (No. S-094-2005 and S-094-2005A) and the Department of Agriculture, Fisheries and Forestry (DAFF) Animal Ethics Committee, Queensland, Australia (No. SA 2008/01/227 and SA 2010/12/339). We used dams and sires of the two subspecies of domestic cow, *Bos taurus taurus* (Angus, A) and *Bos taurus indicus* (Brahman, B), to generate a large number of purebred and reciprocal cross Day 48 embryos (n = 60) and Day 153 fetuses (n = 73) for samples of prenatal tissues. A set of Day 278 calves (term, Day 277–291, n = 17) was delivered by cesarean section for near term placental samples and later used to obtain samples from juveniles at 12–14 months of age. Further information on samples for RNA extraction and cDNA synthesis (see below) is provided in **S1 Table**.

To establish pregnancies for recovery of embryos, fetuses and calves, including placenta, dams were subjected to standard commercial estrous cycle synchronization protocols using Cidirol—Heat Detection and Timed Insemination (HTI) and Cidirol—Timed Insemination (TI) procedures as described previously [101]. All pregnancies were confirmed by ultrasound scanning and embryo, fetal and juvenile tissues obtained after sacrificing animals in an abattoir. All samples were fixed in RNA-later^®^ for 24 hours at 4°C before freezing at -80°C until RNA extraction.

### RNA isolation and reverse transcription

All fetal and juvenile tissue samples and cesarean section placental samples were homogenized with ceramic beads (MoBio Laboratories, Carlsbad, CA, USA) using the Precellys®24 tissue homogenizer (Bertin Technologies, Saint Quentinen Yvelines Cedex, France). Total RNA was extracted using TRI Reagent® (Ambion, Life Technologies™, Inc., Carlsbad, CA, USA) according to the manufacturer’s instructions. Embryonic tissues were homogenized with ceramic beads (MoBio Laboratories, Carlsbad, CA, USA) and the PowerLyzer™ 24 homogenizer (MoBio Laboratories, Carlsbad, CA, USA). Total RNA from embryonic liver and placenta was isolated with TRI Reagent®. Due to small sample size for embryonic brain and heart, AllPrep™ DNA/RNA Micro Kits (Qiagen GmbH, Inc., Hilden, Germany) were used for extraction of nucleic acids. Quantity of RNA was determined by repeated measurements with NanoDrop (ThermoFisher Scientific, Waltham, MA, USA). Quality of RNA was assessed using the Agilent RNA 6000 Nano Kit with a Bioanalyzer 2100 (Agilent Technology Inc., Santa Clara, CA, USA). The mean RNA integrity numbers (RIN) of extracted RNAs from different tissues measured by Bioanalyzer System (Agilent Technologies Inc., Santa Clara, CA, USA) are presented in **S2 Table**.

Complementary DNA (cDNA) was synthesized from 500 ng DNase I (RQ1-DNase, Promega, Madison, WI, USA) treated RNA of each individual tissue sample using SuperScript™ III First-Strand Synthesis System (Invitrogen, Life Technologies™, Inc., Carlsbad, CA, USA) and random hexamer oligonucleotides following the manufacturer’s instructions.

### Target transcript amplification strategy and quantitative real time PCR

**Fig 1:**
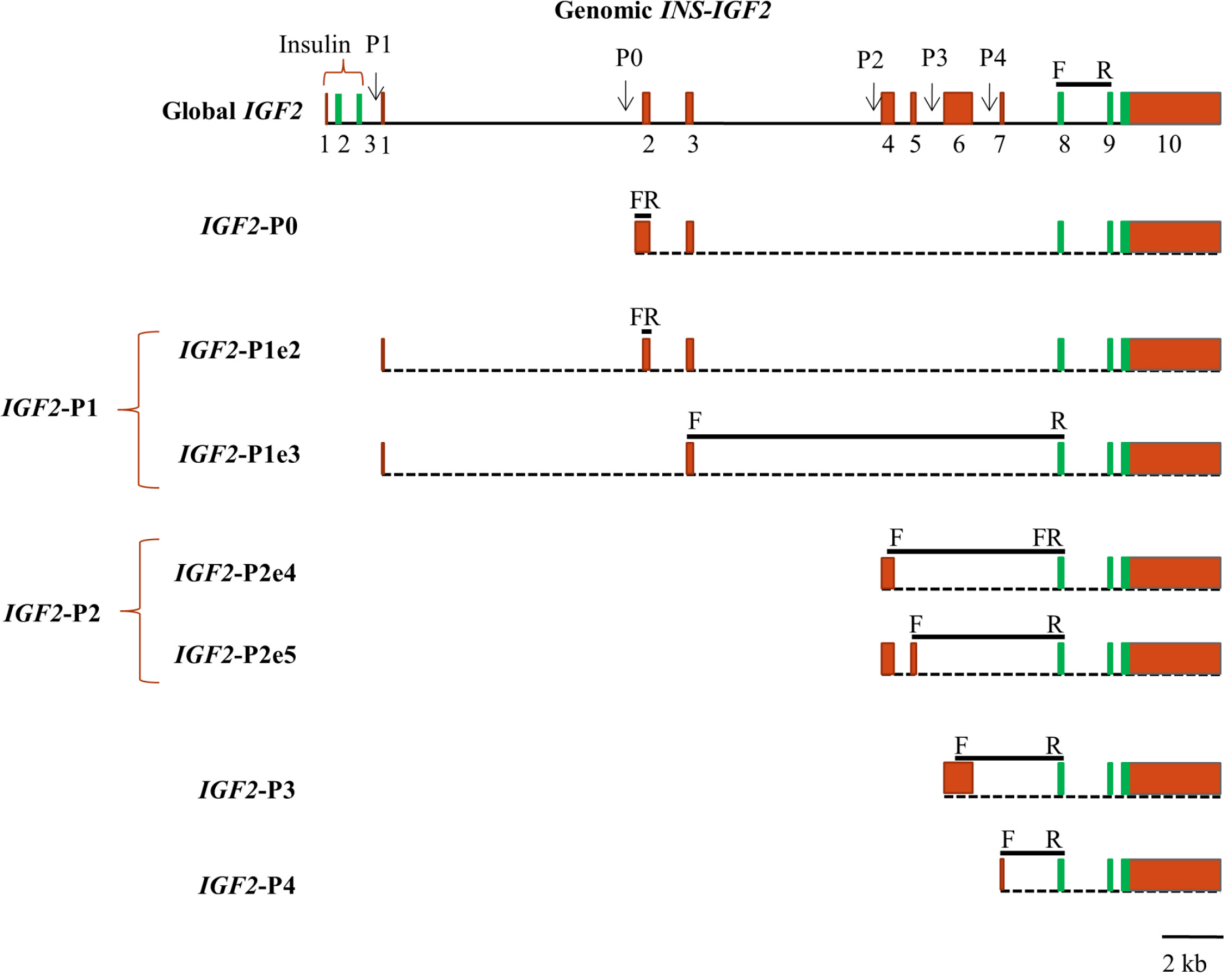
Bovine IGF2 gene and transcript structure with primer locations for amplification of promoter specific transcripts and splice variants. The exon-intron structure of bovine insulin/insulin-like growth factor 2 (*INS/IGF2*, GenBank accession no. EU518675.1) with locations of five promoters (P0, P1, P2, P3 and P4) is shown at the top with promoters (*IGF2*-P0 –P4) and splice variant specific transcripts indicated below. Red and green boxes depict untranslated and protein coding exons, respectively. Forward (F) and reverse (R) primers are indicated with region spanned, including intron where applicable, symbolized by a black bar between primers above the transcript. According to the transcription initiation site of human *IGF2*-P0 transcript, the putative orthologous bovine transcript is predicted to originate from a highly conserved region located upstream of the splice donor site of transcript P1 exon 2. We could specifically amplify bovine *IGF2*-P0 using a strategically designed forward primer within this unique 5’-UTR sequence and the reverse primer located within exon 2. The two splice variants of P1 promoter transcripts include leading exon 1 which is alternatively spliced onto exons 2 and 3 (*IGF2*-P1e2) and exon 3 (*IGF2*-P1e3) plus the coding exons. In order to amplify the P1 promoter transcripts, two pairs of primers located within exon 2 (for *IGF2*-P1e2) and exons 3 and 8 (for *IGF2*-P1e3) were used. This approach was necessary because specific amplification of transcripts derived from P1 promoter failed due to lack of suitable PCR primer sequence in exon 1. Since *IGF2*-transcript P1 exon 2 is part of the first exonic region of transcript *IGF2*-P0, and exon 3 is present in both *IGF2-*P0 and *IGF2-*P1 transcripts, the *IGF2*-P1e2 and -P1e3 amplicons could potentially derive from P0 and/or P1 promoters, depending on tissue and developmental stage. We quantified transcript abundances for two splice variants derived from *IGF2-*P2 promoter which comprise leading exon 4 (*IGF2*-P2e4) or leading exons 4 and 5 (*IGF2*-P2e5) as well as the protein coding exons. The forward primer for *IGF2*-P2e4 was designed to span the junction of exons 4 and 8, and for *IGF2*-P2e5 was in exon 5 with the reverse primer for both splice variants in exon 8. To amplify *IGF2*-P3 and *IGF2*-P4 transcripts, forward primers were designed within exons 6 and 7 with the reverse primer located within exon 8. All primers are detailed in **S3 Table**.

Transcripts quantified included *IGF1* global transcript and the splice variants *IGF1* class 1 and *IGF1* class 2; *IGF2* global transcript and promoter specific transcripts originating from P0, P1 (two transcripts, P1e2 and P1e3), P2 (two splice variants, P2e4 and P2e5), P3 and P4; *IGF1R-* and *IGF2R* transcript, *IR* global transcript and splice variants *IR*-A and *IR*-B; *IGFBP1–8*, as well as *H19* and *AIRN* long noncoding RNA transcripts. Primers were designed to be isoform-specific and span two exons or an exon/intron junction to avoid amplification of genomic DNA sequences. Primer information for all amplicons of target genes is detailed in **S3 Table.** Primer design for promoter and splice variant specific *IGF2* transcripts required extensive in silico analyses in order to be able to assess the complex transcript structure of this gene. Sequences and exon/intron structures for these analyses were retrieved from the literature [16, 43], National Center for Biotechnology Information (NCBI) GenBank database (NCBI reference sequence: AC_000186.1; Gene ID: 281240) and Ensembl project database (ENSBTAG00000013066.5). Since transcripts from P0 promoter were not previously identified in bovine, we performed a sequence similarity search using Basic Local Alignment Search Tool [102] of NCBI, and identified a highly conserved region upstream of bovine *IGF2* exon 2 that corresponded to the human P0 promoter showing (69% homology) [50]. Therefore we hypothesized the existence of a putative orthologous promoter in bovine. An overview of our identification and quantification strategy for *IGF2* transcripts in the context of the genomic structure of *INS/IGF2* (GenBank accession no. EU518675.1) is presented in **Fig 1.**

The first part of this study was designed to systematically measure expression of IGF system components across a broad range of developmental stages and tissues. In light of the fundamental problem to identify stable reference genes across tissues of such rapidly changing developmental stages we opted for a cDNA pooling strategy to assess spatial and temporal differences in expression level. An equal proportion of cDNA from each individual was combined and pooled cDNA used as template in real-time qPCR reactions. The number and sex of individual tissue cDNA samples from different developmental stages used in cDNA pools is summarized in **S1 Table**. An equal proportion of cDNA from all tissue- and developmental stage specific cDNA pools was again pooled to generate a cDNA template for standard curve analysis. The standard curve included 3-fold serial dilutions of initial pooled cDNA template over eight data points. Three replicates were used for each dilution of the cDNA template. Real-time qPCR reactions were performed using Fast Start Universal SYBR Green Master (Roche Diagnostics GmbH, Mannheim, Germany) in an Eppendorf Mastercycler^®^ ep realplex Real-time PCR System (Eppendorf Inc., Hamburg, Germany) following minimum information for publication of quantitative real-time PCR experiments (MIQE) guidelines [103]. The CT (threshold cycle) values of the standards were used to derive a standard curve which shows the CT values as a linear function of natural logarithm of the specified amounts of cDNA. All qPCR reactions were performed in a total volume of 12 μl, containing 6 μl of SYBR master mix, 4 μl of cDNA (equivalent to 12.5 ng of starting RNA), 0.8 μl of primers (5 pmol/μl) and 1.2 μl of double distilled nuclease-free water. PCR was carried out with a 10 minute initial denaturation/activation step at 95°C, followed by 40 cycles of 95°C for 20 seconds, 57–62°C (annealing temperatures, S2 Table) for 30 seconds and 72°C for 20 seconds. Tissue- and developmental stage specific qPCR reactions were performed in triplicate and all investigated tissues and developmental stages were covered in one 96 well plate for each target transcript. Target specificity and integrity was confirmed via sequencing of selected amplicons on a 3730*xl* DNA Analyzer (Applied Biosystems, Inc., Foster City, CA, USA), plots of the melting curve derived by Mastercycler^®^ ep Realplex software (Eppendorf, Inc., Hamburg, Germany), and by electrophoresis of PCR products on a 2% agarose gel (Agarose low EEO, AppliChem GmbH, Darmstadt, Germany) and visual inspection under UV with Gel Doc^TM^ 1000 Single Wavelength Mini-Transilluminator, using Quantity One image analyzing software (Bio-Rad Laboratories, Inc., Hercules, CA, USA) after staining with GelRed™ Nucleic Acid Stain (Biotium, Inc., Hayward, CA, USA). Melt-curve dissociation analyses were performed to ensure that amplifications were free of primer dimers; amplification efficiencies were ≧ 0.9.

The relative abundance of each target transcript was calculated by the relative standard curve method, with determination of PCR amplification efficiency, and expressed in relative units. Transcript abundances are presented in logarithmic scale due to the magnitude of differences between tissues and developmental stages. Since pooled cDNA was used in the quantitative real time RT-PCR reactions for this part of the study, expression data was not normalized using reference genes. We deemed it not appropriate to perform statistical significance tests on technical replicates to compare the average transcript abundances between developmental stages. Rather, we present means and their respective standard deviations from triplicate analyses. Indeed, magnitudes of the vast majority of differences in transcript abundances are such that any statistical testing would have added no meaningful additional information.

### Contribution of *IGF2* promoter specific transcripts to global *IGF2* transcript

In the second part of this study we quantified transcript abundances of IGF2 global and promoter specific transcripts in individual RNA samples of Day 153 fetuses for each of the studied tissues and using Johnson’s Relative Weight procedure determined the contribution of the promoter specific transcripts to global expression [104, 105]. This procedure is based on using individual sample values. It requires normalization against reference genes and provides robust estimates for relative promoter-specific contributions to global transcript, including confidence intervals. Each qPCR experiment for this analysis was performed in duplicate and the mean of both CTs used to calculate the amount of target transcript. An equal proportion of cDNA from all fetuses in each tissue was pooled to generate a cDNA template for standard curve analysis. The standard curve included 2-fold serial dilutions of pooled cDNA template over eight data points, analyzed in triplicate. The relative abundance of each target transcript was automatically calculated by Mastercycler^®^ ep Realplex software (Eppendorf, Inc., Hamburg, Germany) using the relative standard curve method.

We determined expression levels of seven putative reference genes identified by a literature search, including actin beta (*ACTB)*, ribosomal protein S9 (*RPS9*), ubiquitin B (*UBB*), H3 histone family 3A *(H3F3A)*, TATA box binding protein *(TBP)* and vacuolar protein sorting 4 homolog A (*VPS4A*), in each fetal tissue. In placenta, expression level of glyceraldehyde-3-phosphate dehydrogenase (*GAPDH*) was determined instead of *H3F3A*. Details of primers for amplification of reference genes are given in **S4 Table**. As the variation in expression of reference genes frequently differs between tissues, we used NormFinder [106] to identify the most stably expressed genes in each tissue for normalization following recommended procedures. NormFinder uses a model-based approach which enables ranking of reference genes based on stability values, suggesting the best combination of most stably expressed genes. Tissue-specific stability values for all putative reference genes are summarized in **S5 Table**. Expression levels of *IGF2* global and promoter specific transcripts in each tissue were normalized to the geometric mean of the expression levels of identified reference genes [107].

The relative contribution of each promoter specific transcript to global *IGF2* transcript abundance was then calculated by Johnson’s Relative Weight procedure [104, 105] using an SPSS program developed previously [108]. The following linear regression models were used to analyze tissue-specific relative contributions of promoter specific transcripts (P0, P2, P3 and P4) to global *IGF2* expression:

*IGF2* skeletal muscle = *IGF2*-P0 + *IGF2*-P2e4 + *IGF2*-P2e5 + *IGF2*-P3 + *IGF2*-P4

*IGF2* liver = *IGF2*-P2e4 + *IGF2*-P2e5 + *IGF2*-P3 + *IGF2*-P4

*IGF2* placenta,heart,lung,kidney = *IGF2*-P2e5 + *IGF2*-P3 + *IGF2*-P4

where *IGF2* is the relative expression normalized to the reference genes of global *IGF2*, and *IGF2*-P0, *IGF2*-P3 and *IGF2*-P4 are relative expression of the transcripts derived from P0, P3 and P4 promoters, respectively. *IGF2*-P2e4 (transcript with untranslated leader exon 4) and *IGF2*-P2e5 (transcript with untranslated leader exons 4 and 5) are relative expression of the splice variants derived from P2 promoter. Not every equation includes all promoters due to tissue-specific expression.

## Results

### Tissue- and developmental stage specific expression of IGF system components

#### Insulin-like growth factors 1 and 2

**Fig 2:**
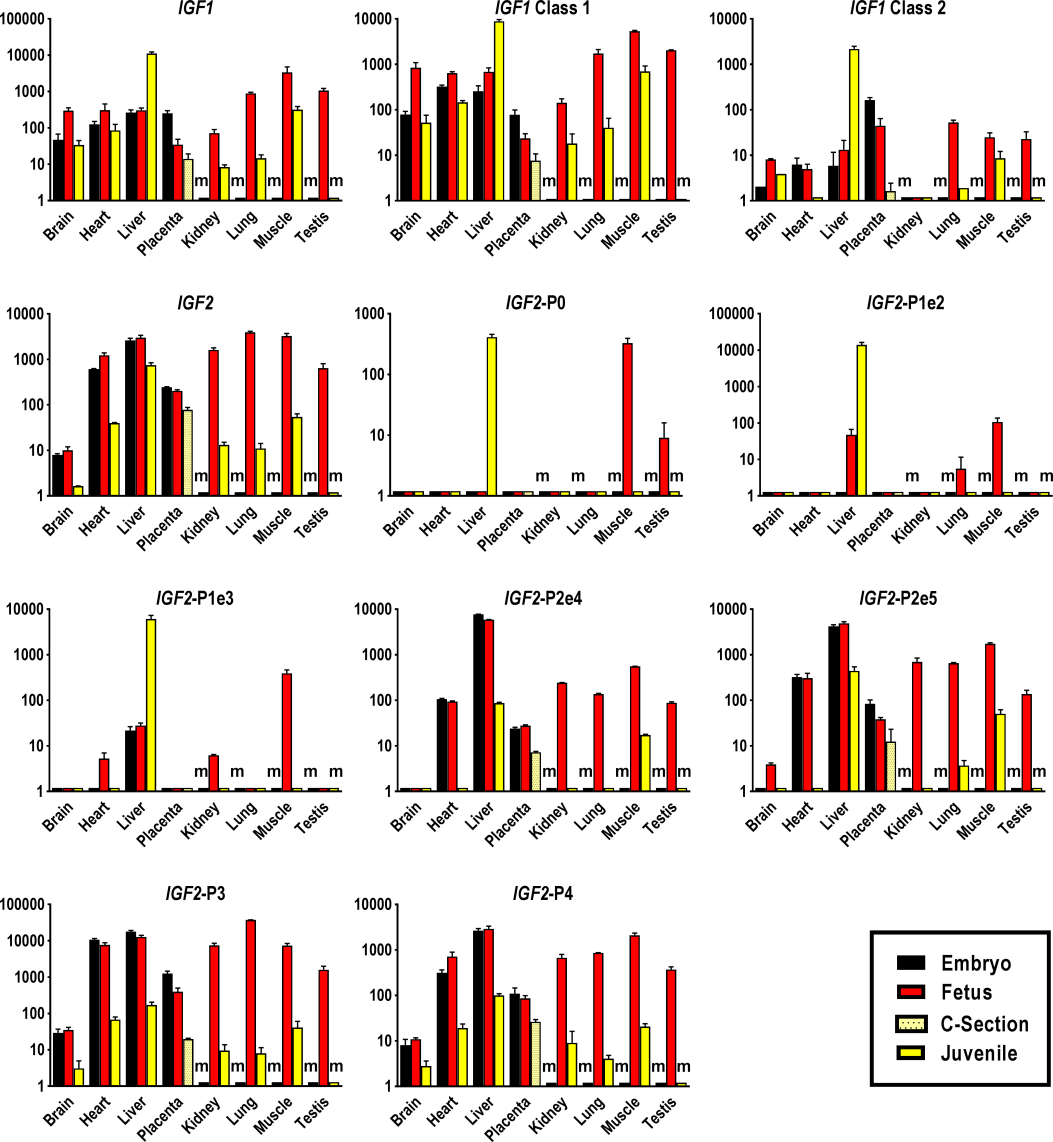
Tissue-specific expression profiles of IGF system ligands in bovine pre- and postnatal developmental stages. Abundances of global *IGF1* transcript and splice variants *IGF1* class 1 and 2, global *IGF2* transcript and promoter and splice variant-specific *IGF2*-P0, *IGF2*-P1e2, *IGF2*-P1e3, *IGF2*-P2e4, *IGF2*-P2e5, *IGF2*-P3 and *IGF2*-P4 transcript were measured in tissues of Day 48 embryos, Day 153 fetuses and 12–14 month-old juveniles. Placental samples were obtained from Day 48 embryos, Day 153 fetuses and term calves born by Caesarean section (C-section) at Day 277/278 of gestation. Means and standard deviations of means for each transcript and tissue were calculated based on triplicate measures of pooled cDNA comprising up to 60 embryonic cDNA samples, 73 fetal cDNA samples, 5 placental cDNA samples of C-section calves and 17 juvenile cDNA samples. Transcript abundances were calculated by the standard curve method and expressed in relative units, and are presented in logarithmic scale. ‘m’ denotes missing tissue such as kidney that is not yet present in embryos, where transcript abundances could not be determined.

Across tissues and developmental stages juvenile liver and fetal skeletal muscle displayed the highest levels of global *IGF1* transcript. Expression in brain and heart peaked at the fetal stage and was lower in most juvenile tissues, with a notable 60.7-fold reduction in lung (**Fig 2 and S6 and S7 Tables**). An exception was juvenile liver, where *IGF1* expression was 36.5-fold higher than in fetal liver (**Fig 2 and S7 Table**). Generally, across tissues and developmental stages, the pattern of *IGF1* class 1 expression was similar to global *IGF1* expression, while *IGF1* class 2 showed a very different pattern. In addition, postnatal increase in liver *IGF1* class 1 and class 2 transcript differed significantly at 13-fold and 165-fold, respectively (**Fig 2**). The highest level of *IGF1* class 2 transcript amongst all prenatal tissues was measured in embryonic placenta, from where it declined towards term (**Fig 2**).

Global *IGF2* transcript levels were highest in embryonic and fetal liver, and fetal lung and skeletal muscle, while liver was the major tissue expressing *IGF2* in juveniles. Brain displayed the lowest expression levels of global *IGF2* transcript across all tissues and developmental stages but expression was still subject to substantial developmental change and 6-fold lower in juveniles as compared to prenatal stages (**Fig 2 and S6 and S7 Tables**). A drastic decline in global *IGF2* expression was evident in juvenile lung where transcript abundance was 355-fold lower as compared to the fetal stage. In contrast, *IGF2* expression in juvenile liver was only 4-fold lower than in fetal liver (**Fig 2 and S7 Table**).

Prenatal expression of transcripts from *IGF2-*P0 promoter was confined to fetal skeletal muscle and testis, with a 30-fold higher abundance in skeletal muscle. Postnatally, the *IGF2*-P0 transcript was only present in liver and at a level comparable to fetal skeletal muscle (**Fig 2**). The *IGF2*-P1e2 amplicon was not detected in embryo tissues, but present in fetal skeletal muscle, lung and liver. In juveniles, it was restricted to liver with 294-fold higher abundance than in the fetus. In the embryo, *IGF2*-P1e3 amplicon was only detected in liver, but by the fetal stage it was also present in heart, kidney and, at the highest level of all prenatal tissues, skeletal muscle. In juveniles this amplicon was again restricted to liver at a level 218-fold higher than at the fetal stage (**Fig 2**).

Comparison of expression patterns of *IGF2*-P2 splice variants revealed that *IGF2*-P2e4 expression was highest in embryonic and fetal liver and 68-fold lower in the juvenile organ. Transcript abundances for *IGF2*-P2e5 followed a similar pattern as *IGF2*-P2e4 except for lung, where it was also detected in juveniles, and for placenta, where it declined from embryo to term (**Fig 2**).

Expression of *IGF2*-P3 promoter transcript was higher in embryonic heart and liver than at the fetal stage. Apart from brain, where expression across developmental stages was very low, this transcript was subject to striking developmental changes. In juveniles, compared to the fetal stage, expression was reduced by 4639-fold in lung, 775-fold in kidney, 179-fold in skeletal muscle, 113-fold in heart and 74-fold in liver. In placenta, expression of *IGF2*-P3 declined throughout gestation with a 20-fold reduction from fetal stage to term (**Fig 2**). The developmental and tissue specific expression pattern of *IGF2*-P4 was similar to *IGF2-*P3 except for fetal lung, where *IGF2-*P4 transcript was expressed at the same level as fetal kidney; both *IGF2-*P3 and *IGF2-*P4 expression patterns were more similar to that of global *IGF2* transcript than any other *IGF2* promoter specific transcript (**Fig 2**).

#### Insulin-like growth factor receptors 1 and 2 and insulin receptor

**Fig 3:**
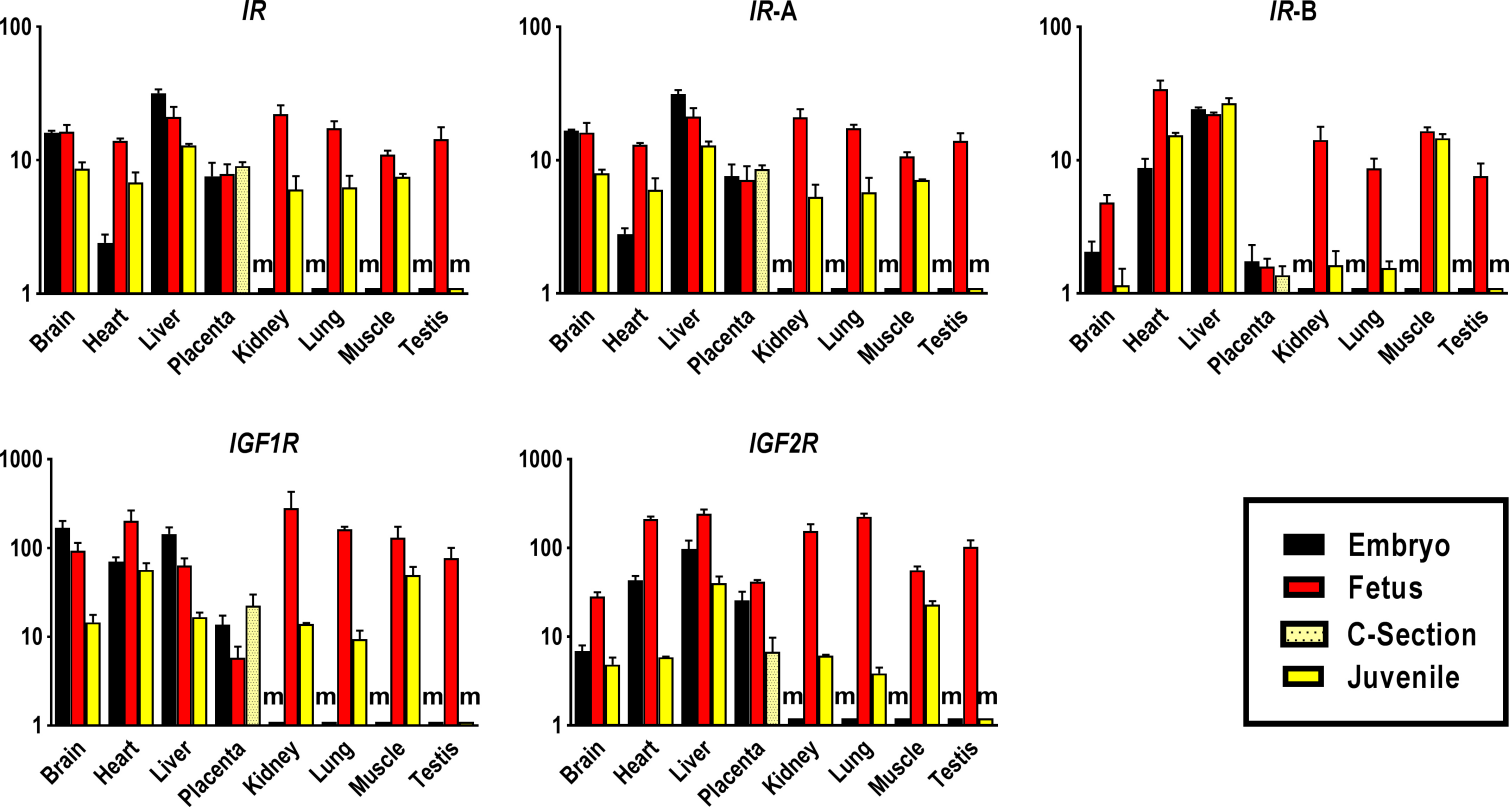
Tissue-specific expression profiles of IGF system receptors in bovine pre- and postnatal developmental stages. Abundances of global *IR* transcript and splice variants *IR*-A and *IR*-B, *IGF1R* and *IGF2R* were measured in tissues of Day 48 embryos, Day 153 fetuses and 12–14 month-old juveniles. Placental samples were obtained from Day 48 embryos, Day 153 fetuses and term calves born by Caesarean section (C-section) at Day 277/278 of gestation. Means and standard deviations of means for each transcript and tissue were calculated based on triplicate measures of pooled cDNA comprising up to 60 embryonic cDNA samples, 73 fetal cDNA samples, 5 placental cDNA samples of C-section calves and 17 juvenile cDNA samples. Transcript abundances were calculated by the standard curve method and expressed in relative units, and are presented in logarithmic scale. ‘m’ denotes missing tissue such as kidney that is not yet present in embryos, where transcript abundances could not be determined.

In comparison to other receptors, *IR* displayed less variation in expression across tissues and developmental stages (**Fig 3 and S6–S8 Tables**). The highest global *IR* transcript levels of all embryonic and postnatal tissues were measured in liver while expression at the fetal stage was highest in skeletal muscle and at similar high levels in heart, kidney, liver, lung and testis. Reduction in postnatal *IR* expression was modest with 3.7-, 2.8- and 1.5-fold lower global transcript levels in juvenile kidney, lung and skeletal muscle than in respective fetal tissues. In placenta, global *IR* transcript abundance remained constant from embryo until term (**Fig 3 and S6–S8 Tables**). The relative temporal-spatial expression pattern for *IR*-A transcript was strikingly similar to global *IR* (**Fig 3**). As compared to the fetal stage, expression of *IR*-A was 2-fold lower in juvenile brain, heart and liver, and 4- and 3-fold lower in postnatal kidney and lung, respectively (**Fig 3**). The *IR*-B transcript displayed a somewhat stronger postnatal decline with 2-, 4-, 6- and 9-fold lower transcript levels in juvenile heart, brain, lung, and kidney, respectively than in fetal tissues. In placenta, *IR*-A and *IR*-B transcript levels were remarkably stable throughout gestation (**Fig 3**).

In brain and liver, *IGF1R* was expressed at similar levels and declined from embryo- to fetal- and juvenile stage. Expression in heart peaked at the fetal stage. In juvenile lung and kidney, transcript levels were 17- and 20-fold lower, respectively, as compared to fetal organs. The highest transcript abundance of *IGF1R* for juveniles was measured in heart and skeletal muscle (**Fig 3 and S7 Table**). Compared to other prenatal tissues, *IGF1R* transcript was less abundant in placenta, where it was lowest at the fetal stage and highest at term (**Fig 3**).

Expression of *IGF2R* transcript was highest in fetuses and lowest in juveniles in all investigated tissues. The increase in expression from embryo to fetal stage ranged from 2.5-fold in liver to 4.9-fold in heart with highest transcript levels observed in fetal heart, liver and lung and lowest levels in brain **(Fig 3 and S6 Table)**. In juveniles, *IGF2R* expression was highest in liver followed by skeletal muscle. Lung revealed the most remarkable postnatal change with a 58.6-fold lower transcript level in juvenile compared to the fetal stage. In contrast, postnatal expression of *IGF2R* in skeletal muscle was only 2.4-fold lower than at the fetal stage **(Fig 3 and S7 Table)**. In placenta, *IGF2R* transcript abundance was 6.2-fold lower at term than at mid-gestation (**Fig 3 and S8 Table**).

#### Insulin-like growth factor binding proteins

**Fig 4:**
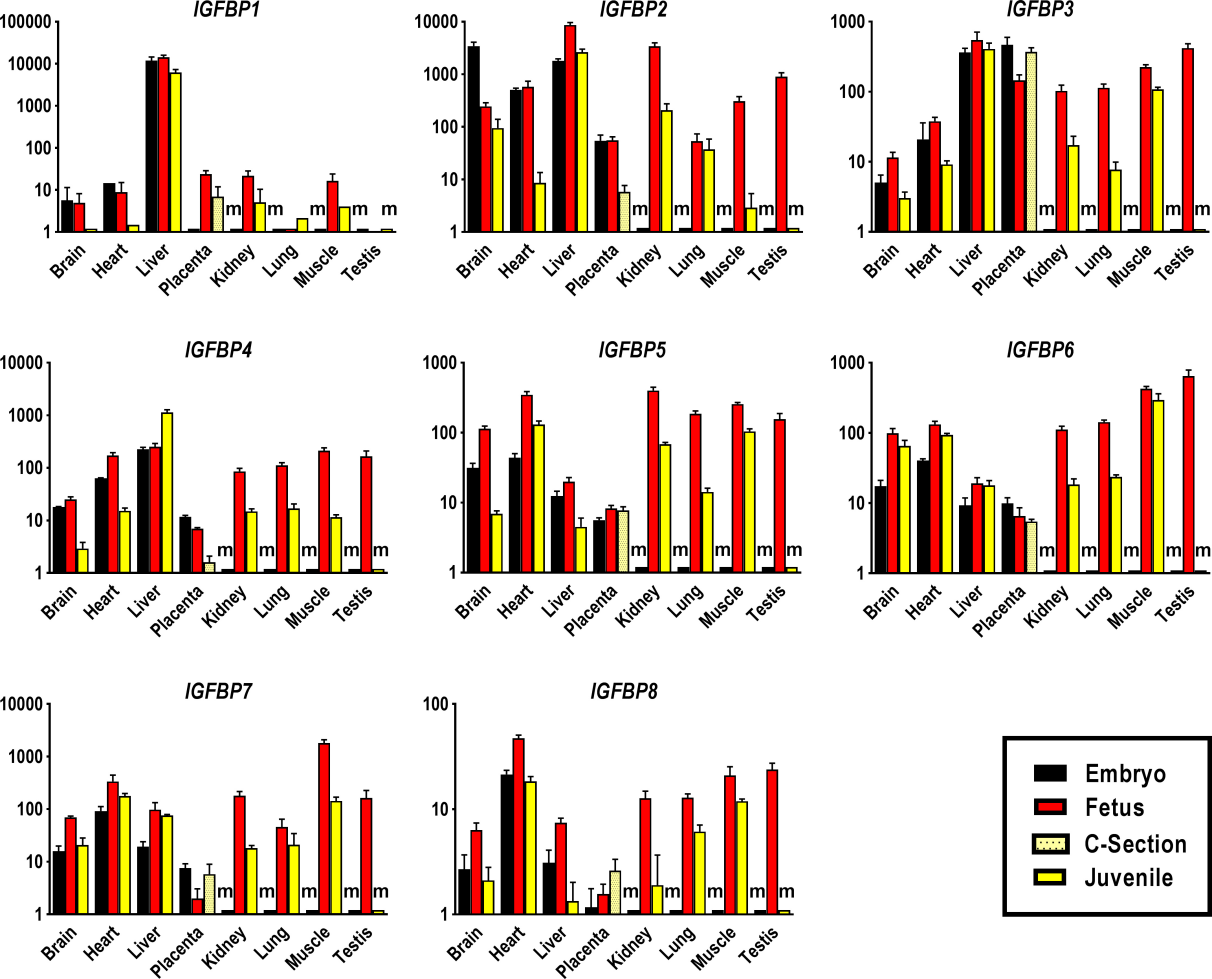
Tissue-specific expression profiles of IGF system binding proteins in bovine pre- and postnatal developmental stages. Abundances of transcripts for *IGFBP1*, *IGFBP2*, *IGFBP3*, *IGFBP4*, *IGFBP5*, *IGFBP6*, *IGFBP7* and *IGFBP8* were measured in tissues of Day 48 embryos, Day 153 fetuses and 12–14 month-old juveniles. Placental samples were obtained from Day 48 embryos, Day 153 fetuses and term calves born by Caesarean section (C-section) at Day 277/278 of gestation. Means and standard deviations of means for each transcript and tissue were calculated based on triplicate measures of pooled cDNA comprising up to 60 embryonic cDNA samples, 73 fetal cDNA samples, 5 placental cDNA samples of C-section calves and 17 juvenile cDNA samples. Transcript abundances were calculated by the standard curve method and expressed in relative units, and are presented in logarithmic scale. ‘m’ denotes missing tissue such as kidney that is not yet present in embryos, where transcript abundances could not be determined.

We analyzed expression patterns of high affinity *IGFBP1* to *6* as well as low affinity *IGFBP7* and *IGFBP8* (**Fig 4**). The IGFBP1 gene displayed a unique expression pattern among *IGFBPs* with almost exclusive expression in liver at all studied developmental stages where transcript abundance in juvenile was 2-fold lower as compared to prenatal stages (**Fig 4, S6 and S7 Tables**). At the embryo stage, *IGFBP2* expression was highest in brain with a 14-fold lower transcript abundance by the fetal stage and only a slight further decline in juvenile (**Fig 4, S6 and S7 Tables**). By the fetal stage, and amongst all sampled tissues, the highest amount of *IGFBP2* transcript was measured in liver. High expression was also detected in kidney, while the lowest amounts of *IGFBP2* transcript were observed in fetal lung and placenta. In juvenile, *IGFBP2* expression was still highest in liver at a level similar to the embryo stage, while transcript abundance in kidney, heart and skeletal muscle were 3–16-, 67- and 106-fold lower than at the fetal stage (**Fig 4 and S7 Table**). Expression of *IGFBP2* was comparatively low and constant in embryonic and fetal placenta, and further declined by 10-fold at term (**Fig 4 and S7 Table**). Expression of *IGFBP3* was highest in liver, where it remained stable across developmental time points, and in placenta and testis. Expression was lower in heart and brain, with a peak at the fetal stage. The 15-fold decline in postnatal expression of *IGFBP3* in lung was higher than in any other tissue (**Fig 4 and S7 Table**). The high level of embryonic placental *IGFBP3* expression was also observed at late gestation (**Fig 4**). The IGFBP4 gene was the only gene from the *IGFBP* family whose expression showed a postnatal organ-specific increase in expression level. Here, transcript abundance in juvenile liver was 4.5–fold higher than at the fetal stage. In other juvenile tissues, transcript abundances were lower than at the fetal stage and at a similar level with a more pronounced 18-fold reduction in skeletal muscle. Brain and placenta exhibited the lowest *IGFBP4* transcript levels at all developmental stages (**Fig 4 and S7 Table**). Expression of *IGFBP5* peaked at the fetal stage in brain, heart and liver and declined in juvenile tissues. The highest transcript levels of *IGFBP5* were measured in fetal heart and kidney. Brain and lung showed the strongest postnatal decline by 16.4- and 13-fold, respectively. In juveniles, *IGFBP5* transcript abundance was highest in heart and skeletal muscle and lowest in brain and liver (**Fig 4 and S7 Table**). Placenta displayed comparatively low and stable expression levels of *IGFBP5* from embryo to term (**Fig 4**). Abundance of *IGFBP6* transcript increased from embryo to fetal stage and was highest in fetal testis and skeletal muscle. Expression remained high in juvenile skeletal muscle and only kidney and lung displayed a 6-fold decline in transcript abundance compared to the fetal stage. Similar to *IGFBP5*, expression of *IGFBP6* was lowest in liver and placenta, but unlike *IGFBP5*, expression did not decline markedly in postnatal brain and liver (**Fig 4 and S7 Table**).

We measured transcript abundances for two low affinity IGFBPs, IGFBP7 and 8. Expression of *IGFBP7* was highest in skeletal muscle and heart and lowest in placenta at all developmental stages. At the fetal stage, transcript abundance in skeletal muscle was substantially higher than in other tissues followed by a 12.7-fold decline in juvenile skeletal muscle. Postnatal expression of *IGFBP7* in kidney also declined by 10-fold (**Fig 4 and S7 Table**). Abundance of *IGFBP8* transcripts was highest in heart and lowest in placenta at all developmental stages. Fetal and juvenile skeletal muscle and fetal testis also showed high levels of *IGFBP8* expression. Expression of *IGFBP8* in juvenile tissues was lower with an approximately 6-fold decline in liver and kidney from the fetal stage (**Fig 4 and S7 Table**).

### Tissue- and developmental stage specific expression of lncRNAs

**Fig 5:**
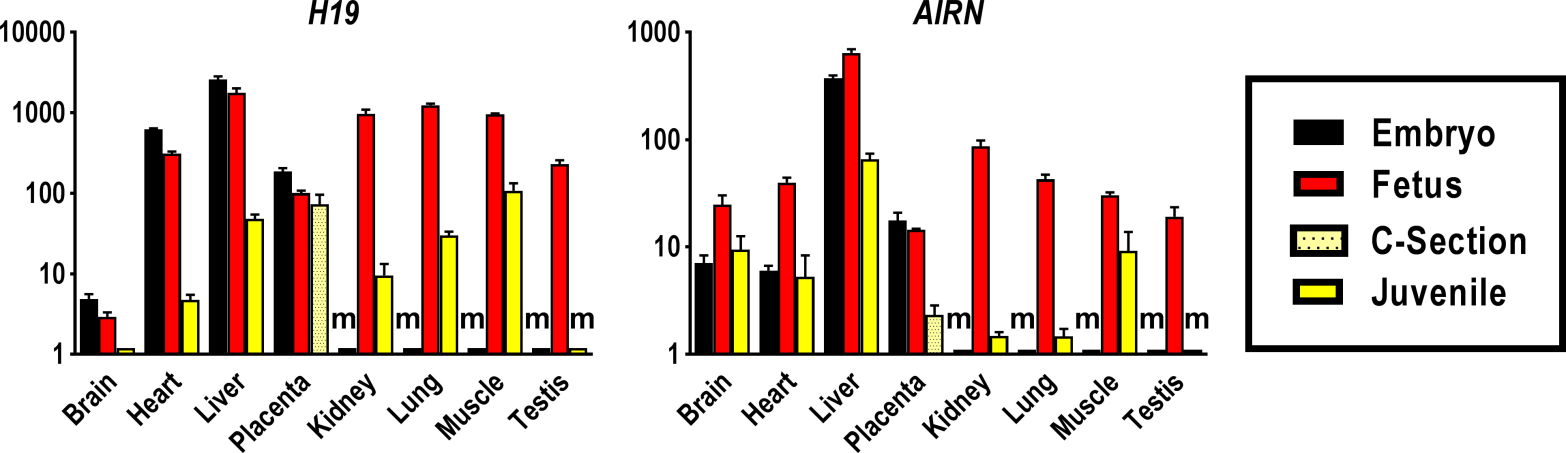
Tissue-specific expression profiles of long non-coding RNAs associated with the IGF system in bovine pre- and postnatal developmental stages. Abundances of *H19* and *AIRN* transcript were measured in tissues of Day 48 embryos, Day 153 fetuses and 12–14 month-old juveniles. Placental samples were obtained from Day 48 embryos, Day 153 fetuses and term calves born by Caesarean section (C-section) at Day 277/278 of gestation. Means and standard deviations of means for each transcript and tissue were calculated based on triplicate measures of pooled cDNA comprising up to 60 embryonic cDNA samples, 73 fetal cDNA samples, 5 placental cDNA samples of C-section calves and 17 juvenile cDNA samples. Transcript abundances were calculated by the standard curve method and expressed in relative units, and are presented in logarithmic scale. ‘m’ denotes missing tissue such as kidney that is not yet present in embryos, where transcript abundances could not be determined.

We investigated expression of the two imprinted long non-coding RNA genes associated with the IGF system, *H19* and *AIRN*. Across all tissues, expression of *H19* was highest in the embryo, declined slightly by the fetal stage and was substantially lower in juvenile (**Fig 5, S6 and S7 Tables**). Embryonic and fetal liver displayed the highest, and brain the lowest, *H19* transcript levels of all prenatal tissues. Similar and comparatively high levels of *H19* transcript were found in fetal kidney, lung and skeletal muscle with somewhat lower levels in heart and testis. Kidney displayed the strongest decline in postnatal *H19* expression with more than 100-fold lower transcript abundance as compared to the fetal stage. Amongst postnatal tissues, *H19* transcript level was highest in skeletal muscle which displayed a 9-fold reduction from fetal to juvenile stage. Expression of *H19* in placenta declined 2-fold from embryo to fetal stage and was unchanged at term (**Fig 5, S7 and S8 Tables**).

Expression of *AIRN* transcript increased from embryo to fetal stage and declined in juvenile tissues (**Fig 5, S6 and S7 Tables**). Across developmental stages *AIRN* transcript was most abundant in liver with 10-fold lower expression in juvenile. Expression was also high in fetal kidney and lung, which showed the strongest decline in transcript abundance with 58- and 29-fold lower transcript levels in juvenile. Brain and heart displayed similar *AIRN* expression levels at the embryo and juvenile stages and brain, heart and skeletal muscle had a similar, milder reduction in expression level in juvenile as compared to fetal tissues (**Fig 5, S7 Table**). Placental expression of *AIRN* was similar at the embryo and fetal stage, but 6-fold lower at term (**Fig 5 and S8 Table**).

### Contribution of promoter specific *IGF2* transcripts to global *IGF2* transcript abundance in fetal tissues

**Fig 6:**
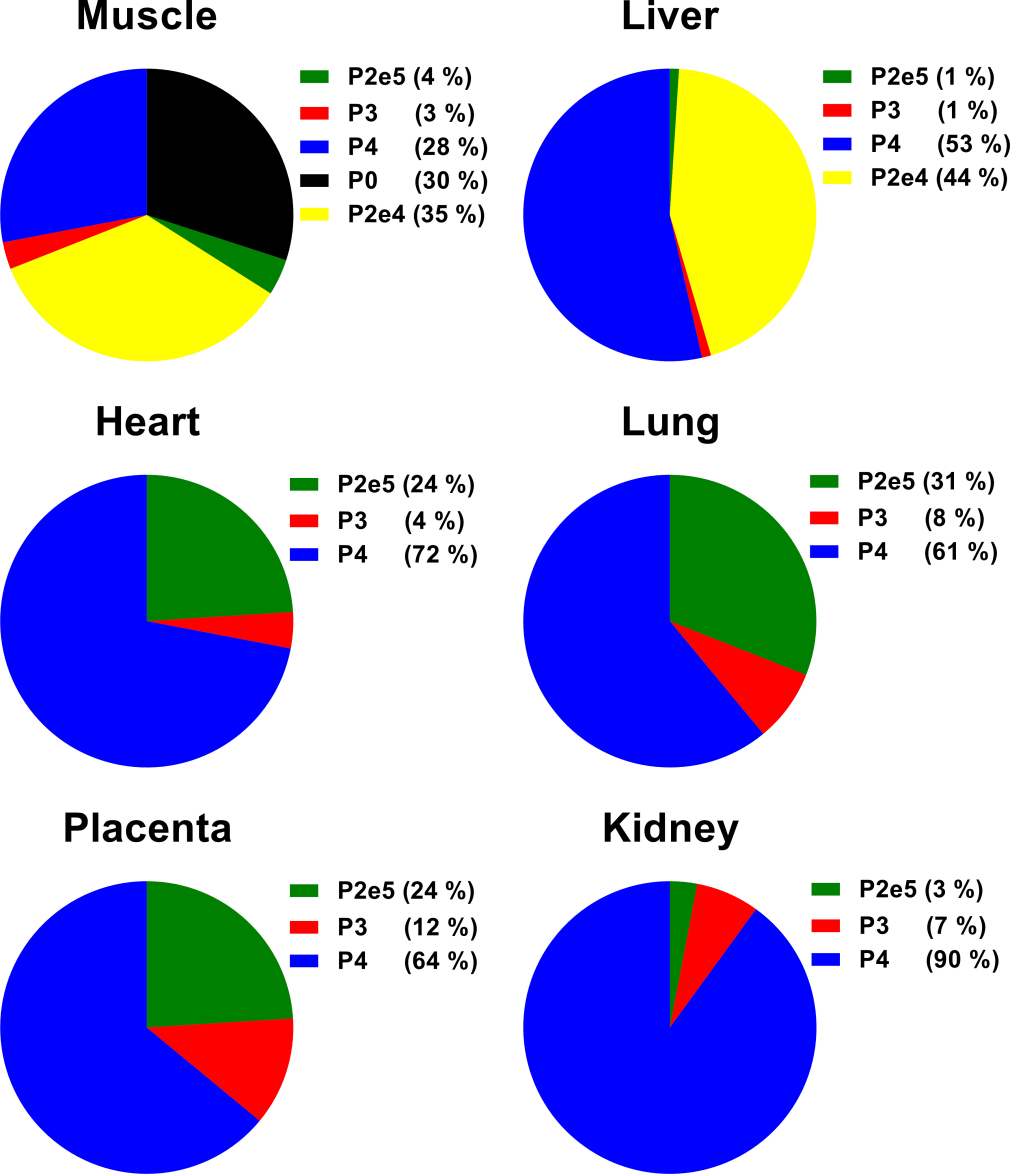
Relative contribution of promoter and splice variant-specific *IGF2* transcripts to global *IGF2* transcript abundance in fetal tissues and placenta. *IGF2*-P0, *IGF2*-P3 and *IGF2*-P4 are percent transcript abundance derived from P0, P3 and P4 promoters, respectively. Splice variants of promoter P2 transcript are *IGF2*-P2e4 with untranslated leader exon 4 and *IGF2*-P2e5 with untranslated leader exons 4 and 5. Estimated means are from 73 fetal cDNA samples per tissue and 95% confidence intervals are detailed in **S9 Table**. Transcript abundances were calculated by the standard curve method, normalized with reference genes and expressed in relative units. The relative contribution of each promoter-specific transcript to global *IGF2* transcript abundance was calculated by Johnson’s Relative Weight procedure.

We determined for the first time the contribution of promoter-specific *IGF2* transcripts (**Figs 1 and 2**) to global *IGF2* transcript in fetal tissues including placenta by multiple regression analysis (**Fig 6**). Estimated means with 95% confidence intervals are presented in **S9 Table**. Contributions of 53%, 61%, 72% and 90% to global *IGF2* transcript identified *IGF2-*P4 as the predominant promoter in bovine fetal liver, lung, heart and kidney, respectively. This promoter was also responsible for 64% of all *IGF2* transcripts measured in placenta. Amongst all tissues studied, *IGF2-*P4 was least active in skeletal muscle but it still contributed 28% of global transcript. The second most abundant transcript in fetal lung, heart and placenta was *IGF2*-P2 derived *IGF2*-P2e5, accounting for 31%, 24%, and 24% of global transcript, respectively. However in fetal liver and skeletal muscle, the alternative splice variant derived from *IGF2-*P2 promoter, *IGF2*-P2e4 transcript, dominated and accounted for 44% and 35% of global *IGF2* expression, respectively. Although *IGF2*-P0 accounted for 30% of global *IGF2* and was therefore one of the most common *IGF2* transcripts in muscle, it did not contribute to *IGF2* expression in any other fetal tissue or placenta. Promotor *IGF2-*P3 was active at low levels with contributions of 1–8% in fetal tissues and 12% in placenta.

## Discussion

We provide here an atlas of tissue- and developmental stage specific gene expression for the bovine insulin-like growth factor (IGF) system, including imprinted long non-coding (lnc) RNAs H19 and AIRN. This mammalian IGF expression catalogue informs basic and comparative IGF research and provides reference data for an important agricultural species and biomedical model.

Our comprehensive profile of expression patterns and comparisons of pre- and postnatal changes in expression of IGF ligands support established roles of *IGF1* in growth and development [109]. Across developmental stages and tissues, global *IGF1* expression was highest in juvenile liver, consistent with data from sheep [31, 110], mouse [111] and rat [112–114]. However, *IGF1* expression in all other tissues peaked at the fetal stage, a clear indication of the significant role of IGF1 in mammalian prenatal growth [2, 97, 115, 116]. As reported previously [117], expression was highest in fetal muscle where IGF1 has protein anabolic effects [118]. The expression pattern for *IGF1* class 1 transcript was more similar to *IGF1* global transcript, suggesting that class 1 is the predominant transcript across tissues and developmental stages. In skeletal muscle and liver of the third trimester sheep fetus, IGF1 class 1 transcript was also much more abundant than class 2 transcript [97]. Expression of *IGF1* class 2 transcript displayed considerable developmental stage and tissue specificity, with strongest expression in postnatal liver. Stronger postnatal hepatic expression of *IGF1* class 2 compared to class 1 transcript could be due to greater dependency of this transcript on growth hormone as shown in sheep [110]. Our data thus extends previous limited information on *IGF1* class 1 and 2 transcript expression in pre- and postnatal tissues of cattle [119, 120], sheep [97, 110, 121], pig [122, 123], mouse [124] and rat [125] to earlier embryo-fetal stages.

We found that expression of *IGF2* was broadly similar in embryonic and fetal bovine tissues, but much lower in juvenile tissues. Postnatal decline in expression of global *IGF2* transcript was considerably stronger than for *IGF1*, highlighting the special role of *IGF2* in prenatal growth and development described previously [126–128]. Significant downregulation of *IGF2* after birth has been reported for cattle, sheep and human [90–92, 98, 129]. In rat, *IGF2* transcripts were undetectable by Northern blot in all adult tissues except brain, spinal cord and striated muscle [13, 130, 131]. Postnatal decline in *IGF2* expression was least in liver, the major source of circulating IGF2 in adults [8] and consistent with considerable IGF2 levels in adult cattle [132]. The higher expression of *IGF2* in postnatal liver compared to other tissues may be caused by tissue-specific relaxation of *IGF2* imprinting or a change from imprinted to non-imprinted promoter use as previously demonstrated in human [37, 133]. The exclusive expression of *IGF2*-P0 transcript in juvenile liver and high levels of *IGF2*-P1e2 and -P1e3 transcripts in this tissue may reflect a combined imprinted/non-imprinted promoter scenario. In human, P0 transcript is expressed from the paternal allele while P1 transcripts show biallelic expression at all developmental stages [37, 50].

Exclusive expression of *IGF2*-P0 transcript in bovine fetal skeletal muscle and testis, is in agreement with reported *IGF2*-P0 expression in human fetal skeletal muscle [50]. To our knowledge fetal testis has not yet been tested for expression of *IGF2*-P0 transcript in human or any other species. Human and bovine thus indicate an evolutionary shift in *IGF2* expression from mouse, where *Igf2*-P0 transcript is confined to placenta [127]. We also demonstrate for the first time a developmental shift in tissue-specificity of *IGF2*-P0, which is no longer active in juvenile bovine skeletal muscle but active in juvenile liver. This contrasts with ubiquitous *IGF2*-P0 expression in adult human tissues [50] although some of these differences may be explained by the different developmental age of bovine tissues studied.

Splice variants of *IGF2-*P1 transcripts including exon 1, which is alternatively spliced onto exons 2 and/or 3 as well as the coding exons, were previously observed in bovine [16], pig [44], human [134, 135] and sheep [42]. Considering sequence based restrictions of our *IGF2* promoter 1-specific transcript amplification strategy described in **Fig 1,** and our finding that *IGF2*-P0 transcript is the predominant transcript in bovine fetal skeletal muscle, *IGF2-*P1e2 and *IGF2-*P1e3 transcripts detected in this tissue could potentially originate from the *IGF2-*P0 promoter. However, we conclude that *IGF2-*P1e2 and *IGF2-*P1e3 transcripts in liver, whose abundance increased in postnatal tissue, predominantly derive from *IGF2*-P1 promoter activity. This is based on an *IGF2*-P0 expression level in juvenile liver similar to that in fetal skeletal muscle and the fact that relative transcript abundance of *IGF2*-P1e2 and *IGF2*-P1e3 in juvenile liver was 130-fold and 15-fold higher than in fetal muscle. Our demonstration of increased activity of *IGF2-*P1 promoter in postnatal liver where *IGF2-*P2, *IGF2-*P3 and *IGF2-*P4 activity decreases, is consistent with the developmental shift in promoter activity reported for human [46, 50], pig [44], sheep [42, 91] and bovine [92].

Temporal-spatial expression patterns of the two splice variants that originate from *IGF2*-P2 promoter, *IGF2*-P2e4 and P2e5, showed some similarities (**Fig 2**), but quantitative analyses of promoter-specific transcripts in fetal tissues revealed that only *IGF2*-P2e4 variant is a major contributor to *IGF2* transcript abundance in fetal liver and skeletal muscle (**Fig 6**). This suggests that *IGF2-P2e4* could be actively involved in the production of endocrine IGF2 in the bovine fetus.

Analysis of contributions of promoter specific *IGF2* transcripts to global *IGF2* transcript expression revealed that *IGF2*-P3 and *IGF2*-P4 are the least and most active promoters, respectively, in all bovine fetal tissues except kidney. This is in agreement with data from fetal mouse and rat, where *IGF2-*P3, which is orthologous to bovine *IGF2-*P4 promoter, was most active [48], but differs somewhat from human where *IGF2-*P3 and *IGF2-*P4, both orthologues of the respective bovine promoters, were active at high and moderate levels [136].

Amongst receptors studied, relative spatio-temporal expression patterns for global *IR* and splice variant *IR-*A were more stable than those for *IGF1R* and *IGF2R*. This may be explained by predominant involvement of insulin receptors in metabolic pathways, rather than in engendering growth responses [137]. Mice without *Insr* display virtually unimpaired prenatal development with only slight reduction in birth weight [70] and catastrophic loss of metabolic control only after birth [7, 138]. The similarity in expression patterns of *IR* global transcript and *IR*-A suggests that *IR*-A is the predominant isoform of insulin receptor expressed in tissues. The most notable change, and consistent with other relative changes in expression patterns in the IGF system, was the decline in postnatal *IR*-B transcript in kidney and lung.

The similarity in expression patterns of *IGF2R* and *IGF2* is consistent with the crucial role of *IGF2R* for normal development [139]. Observed concomitant downregulation of *IGF2R* and *IGF2* could be expected from the essential regulatory function of IGF2R for IGF2 bioavailability [7, 61, 96, 140]. Evidence for active signaling of IGF2 through IGF2R has been reported and several lines of evidence suggest that IGF2 stimulates trophoblast migration through IGF2R [72, 141, 142]. This is consistent with the stable expression of *IGF2* and *IGF2R* in placenta from embryo to fetal stage and the decline in both transcripts at term in the present study.

Transcript abundances for IGFBPs revealed exclusive strong *IGFBP1* expression in liver across all developmental stages. A similar expression pattern has been reported for fetal liver in mouse and human [143, 144] and fetal and adult liver in rat [145, 146] and reflects the endocrine role of IGFBP1 [147–150]. The postnatal decrease in expression of *IGFBP2* in skeletal muscle, heart and kidney suggests a significant role before birth. This is supported by higher circulating IGFBP2 levels in the fetus as compared to adults in a number of species including rat, human, pig and rhesus monkey [99, 151–154]. The relative expression pattern of *IGFBP3* in postnatal tissues showed similarity to the pattern observed for postnatal *IGF1* expression. In the sheep fetus, circulating IGFBP3 levels are positively correlated with IGF1 [117, 155]. Expression of *IGFBP3* in placenta remained high throughout gestation. In human and rhesus monkey placenta *IGFBP3* is co-expressed with *IGF2* and has been proposed to modulate IGF2 effects in an autocrine/paracrine fashion [156, 157]. The IGFBP4 gene stands out as the only binding protein gene that displayed an increase in transcript abundance in a juvenile tissue, i.e., liver. This is consistent with increased *IGFBP4* expression in neonatal pig liver [100] and increased circulating IGFBP4 in sheep after birth [155]. It is possible that the postnatal increase in hepatic *IGFBP4* expression is associated with increased expression of *IGF1*. It has been demonstrated that IGFBP4 enhances growth stimulatory effects of endocrine IGF1 by increasing bioavailability of IGF1 via an IGFBP4 protease-dependent mechanism [158]. High levels of *IGFBP6* expression in fetal and juvenile bovine skeletal muscle may be explained by the involvement of this gene in inhibiting IGF2-induced proliferation and differentiation of myoblasts [159]. Similarly, high level of *IGFBP7* expression in bovine fetal skeletal muscle may also be associated with a potential role in inhibiting myoblast differentiation [160, 161].

The most pronounced changes in transcript levels from pre- to postnatal stages were evident in lncRNAs, consistent with the fundamental roles of this RNA class in growth and development [162–165]. Substantially greater spatio-temporal changes in expression of *H19* as compared to *AIRN* highlights the pivotal functions of H19 RNA as source of regulatory miRNA that impact *IGF1R* and Smad transcription factors [57, 166] and thus contribute to its role as a major regulator of an imprinted gene network [167] that controls growth [168]. Across tissues and for each developmental stage, relative expression patterns for *H19* were highly similar to those for *IGF2*, which is consistent with current models for coordinated regulation of both genes [169–171]. Cell lineage dependent *H19* expression has been described in sheep [172] and the human fetus [173] where, as in the present study, transcript abundance was lowest in brain. We found that expression of *H19* in bovine fetal kidney, lung and liver was at similar high levels or higher than in fetal skeletal muscle, where it was originally described as a major regulator of prenatal growth and differentiation [168]; whether *H19* has a similar role in these tissues remains to be elucidated. Expression of *H19* in juvenile tissues was much lower than at prenatal stages, except for skeletal muscle, where significant transcription persists. This suggests that growth regulatory functions of H19 in bovine prenatal muscle [168] may continue well into postnatal development. High levels of *H19* RNA have also been found in postnatal skeletal muscle of mouse, where H19 encoded miRNAs promote differentiation and regeneration [166].

In light of observed similarities in *H19/IGF2* expression patterns, the different spatio-temporal patterns for *AIRN* and *IGF2R* transcript abundances are somewhat unexpected. Considering the antisense nature of *AIRN/IGF2R* transcripts and their mutually exclusive, interdependent mode of expression in mouse, where AIRN RNA silences *IGF2R* [81, 174, 175] an inverse relationship could have been expected. The seemingly unrelated expression patterns may indicate species differences and/or further, possibly organ-specific, roles of *AIRN* in silencing additional imprinted genes [175] or involvement in trans-regulatory processes similar to those identified for *H19* [165].

Lung and kidney showed the highest and liver the lowest relative postnatal reduction in expression of IGF system components. This may be explained by additional prenatal functions of lung and kidney as the flow of fluid from the fetal lung and bladder are major contributors to amniotic fluid [176, 177]. Interestingly, amniotic fluid is a significant source of IGFs [178] as large amounts of fluid are swallowed by the fetus [179] and growth factors cross the gut to enter systemic circulation [180]. The lesser changes in postnatal liver likely reflect the continuing role of this organ as a major source of circulating IGF system components [8, 181–183].

To our knowledge, this is the first comprehensive study in any species to investigate changes in expression of IGF system components and their major regulatory lncRNAs across tissues and developmental stages using real time qPCR. Our expression atlas for the bovine insulin-like growth factor system provides important reference data for future studies of the mammalian IGF system. This includes dissection of prenatal effects on postnatal phenotype where IGF system components and epigenetic mechanisms regulating them, including imprinting and miRNAs, appear to be major programming components [181, 184–187].

## Supporting information

S1 Table Number and sex of individuals used for developmental stage and tissue-specific cDNA pools. (DOCX)

S2 Table RNA integrity number (RIN) of RNA extracted from different tissues. (DOCX)

S3 Table Details of forward (F) and reverse (R) primers used for amplification of transcripts of target genes. (DOCX)

S4 Table Details of forward (F) and reverse (R) primers used for amplification of transcripts of reference genes. (DOCX)

S5 Table Stability values for studied reference genes and best combination of genes for different tissues as calculated by NormFinder. (DOCX)

S6 Table Comparison of changes in gene expression in the bovine IGF system between Day 48 embryo and Day 153 fetal stages. (DOCX)

S7 Table Comparison of changes in gene expression in the bovine IGF system between Day 153 fetal and 12–14 month juvenile stages. (DOCX)

S8 Table Comparison of changes in gene expression in the bovine IGF system of placenta from embryo stage to term. (DOCX)

S9 Table Estimated relative contributions of *IGF2* promoter-specific transcripts to global IGF2 transcript abundance with 95% confidence intervals for each fetal tissue. (DOCX)
