# Supplementary material for: Atlas of tissue- and developmental stage specific gene expression for the bovine insulin-like growth factor (IGF) system
Source: PLoS One. 2018 Jul 12;13(7):e0200466. doi: 10.1371/journal.pone.0200466 (PMC6042742; doi:10.1371/journal.pone.0200466)
Supplement: S1 Table — (DOCX) [file pone.0200466.s001.docx]

**S1 Table.** **Number and sex of individuals used for developmental stage and tissue-specific cDNA pools^a^.**

|  | **n** | **Liver** | **Brain^b^** | **Heart** | **Placenta^c^** | **Lung** | **Kidney** | **Muscle^d^** | **Testis** |
| --- | --- | --- | --- | --- | --- | --- | --- | --- | --- |
| **Embryo** | Total | 60 | 60 | 60 | 60 | _ | _ | _ | _ |
|  | Male | 29 | 29 | 29 | 29 | _ | _ | _ | _ |
|  | Female | 31 | 31 | 31 | 31 | _ | _ | _ | _ |
| **Fetus** | Total | 73 | 73 | 73 | 73 | 73 | 73 | 73 |  |
|  | Male | 27 | 27 | 27 | 27 | 27 | 27 | 27 | 27 |
|  | Female | 46 | 46 | 46 | 46 | 46 | 46 | 46 |  |
| **C-section**  **calf** | Total | _ | _ | _ | 5 | _ | _ | _ | _ |
|  | Male | _ | _ | _ | 2 | _ | _ | _ | _ |
|  | Female | _ | _ | _ | 3 | _ | _ | _ | _ |
| **Juvenile** | Total | 17 | 16 | 17 | _ | 17 | 17 | 17 | _ |
|  | Male | 10 | 10 | 10 | _ | 10 | 10 | 10 | _ |
|  | Female | 7 | 6 | 7 | _ | 7 | 7 | 7 | _ |

^a^ – indicates tissue was not available, i.e., not yet developed in embryo (lung, kidney, muscle, testis) or not available for juvenile (placenta) and castrated juvenile male (testis). ^b^ Telencephalon. ^c^ Cotyledon from a large placentome close to the embryo and cotyledon from a large placentome close to the fetus. ^d^ *M. semitendinosus*.
