# Supplementary material for: Atlas of tissue- and developmental stage specific gene expression for the bovine insulin-like growth factor (IGF) system
Source: PLoS One. 2018 Jul 12;13(7):e0200466. doi: 10.1371/journal.pone.0200466 (PMC6042742; doi:10.1371/journal.pone.0200466)
Supplement: S2 Table — (DOCX) [file pone.0200466.s002.docx]

**S2 Table.** **RNA integrity number (RIN) of RNA extracted from different tissues.** Means, standard errors of means and number of RNA samples (n) selected for quality control purposes are shown^a^.

|  | **Muscle^b^** | **Lung** | **Kidney** | **Heart** | **Liver** | **Brain^c^** | **Placenta^d^** | **Testis** |
| --- | --- | --- | --- | --- | --- | --- | --- | --- |
| **Embryo** | _ | _ | _ | 9.00±0.55 (n=28) | 8.93±0.44 (n=35) | 8.60±0.40 (n=38) | 6.35±1.44 (n=59) | _ |
|  |  |  |  |  |  |  |  |  |
| **Fetus** | 8.21±0.41 (n=73) | 8.85±1.37 (n=49) | 7.41±1.69 (n=48) | 8.45±0.40 (n=45) | 8.05±0.45 (n=73) | 8.38±0.45 (n=56 ) | 7.16±1.26 (n=51) | 5.85±1.3 (n=24) |
|  |  |  |  |  |  |  |  |  |
| **C-section calf** | _ | _ | _ | _ | _ | _ | 4.36±1.21 (n=5 ) | _ |
| **Juvenile** | 7.16±0.77 (n=11 ) | 7.43±0.76 (n=12 ) | 4.16±1.72 (n=10 ) | 7.67±0.17 (n=9 ) | 7.54±0.67 (n=9 ) | 6.74±0.48 (n=10 ) | _ | _ |

^a^ – denotes tissue not available, i.e., not yet developed in embryo (Lung, kidney, muscle, testis) or not available for juvenile (placenta) and castrated juvenile male (testis). ^b^ *M. semitendinosus*. ^c^ Telencephalon. ^d^ Cotyledon from a large placentome close to the embryo or fetus.
