# Supplementary material for: Atlas of tissue- and developmental stage specific gene expression for the bovine insulin-like growth factor (IGF) system
Source: PLoS One. 2018 Jul 12;13(7):e0200466. doi: 10.1371/journal.pone.0200466 (PMC6042742; doi:10.1371/journal.pone.0200466)
Supplement: S3 Table — (DOCX) [file pone.0200466.s003.docx]

**S3 Table. Details of forward (F) and reverse (R) primers used for amplification of transcripts of target genes.** Primer sequences, annealing temperatures (AT), amplified fragment length (FL, in base pairs, bp), location of primers in the target gene (Exon/Intron) and GenBank accession number are shown.

| **Target transcript** | **Primer sequence (5’ to 3’)** | **AT (°C)** | **FL (bp)** | **Exon (E)/Intron (I)** | **Accession No.** |
| --- | --- | --- | --- | --- | --- |
| *IGF1* (F) | GATGCTCTCCAGTTCGTGTGC | 60 | 140 | E-2 | NW_003103925.1 |
| *IGF1* (R) | TCCAGCCTCCTCAGATCACAG | 60 | 140 | E-3 | NW_003103925.1 |
|  |  |  |  |  |  |
| *IGF1* Class1 (F) | TTCAGAAGCAATGGGAAAAATCAG | 59 | 115 | E-1 | NW_003103925.1 |
| *IGF1* Class1 (R) | ATAGAAGAGATGCGAGGAGGATGTG | 59 | 115 | E-2 | NW_003103925.1 |
|  |  |  |  |  |  |
| *IGF1* Class2 (F) | TCATAATACCCACCCTGACCTGC | 59 | 105 | E-1,2 | NW_003103925.1 |
| *IGF1* Class2 (R) | ATAGAAGAGATGCGAGGAGGATGTG | 59 | 105 | E-2 | NW_003103925.1 |
|  |  |  |  |  |  |
| *IGF2* (F) | CTTCGCCTCGTGCTGCTATG | 60 | 134 | E-8 | NM_174087.3 |
| *IGF2* (R) | GTCGGTTTATGCGGCTGGAT | 60 | 134 | E-9 | NM_174087.3 |
|  |  |  |  |  |  |
| *IGF2*-P0 (F) | CACGCTCTAAAAATGCCCTTCA | 60 | 103 | I-1 | EU518675.1 |
| *IGF2*-P0 (R) | TGCTCTGGCTGTGGTGCTCA | 60 | 103 | E-2 | EU518675.1 |
|  |  |  |  |  |  |
| *IGF2*-P1e2 (F) | CCTCAGCCTCATCCCCTCCTTTGC | 60 | 217 | E-2 | EU518675.1 |
| *IGF2*-P1e2 (R) | CTGTGCTCTATTTGCTGTGTTGTCT | 60 | 217 | E-2 | EU518675.1 |
|  |  |  |  |  |  |
| *IGF2*-P1e3 (F) | GGTCAGCCCTTTGCCCAG | 62 | 179 | E-3 | NM_174087.3 |
| *IGF2*-P1e3 (R) | CACCAGCACCGACTTTCCT | 62 | 179 | E-8 | NM_174087.3 |
|  |  |  |  |  |  |
| *IGF2*-P2e4 (F) | TCCAGCCTCGCGACATCA | 61 | 66 | E-4,8 | DQ298749.1 |
| *IGF2*-P2e4 (R) | CAAGAAGGCAAGAAGCACCA | 61 | 66 | E-8 | DQ298749.1 |
|  |  |  |  |  |  |
| *IGF2*-P2e5 (F) | TACGCAAGTCCAACGCATAGA | 60 | 160 | E-5 | DQ298745.1 |
| *IGF2*-P2e5 (R) | CAAGAAGGCAAGAAGCACCA | 60 | 160 | E-8 | DQ298745.1 |
|  |  |  |  |  |  |
| *IGF2*-P3 (F) | AGACAGCCCGTCCTCCCTA | 60 | 246 | E-6 | BC116039.1 |
| *IGF2*-P3 (R) | CACCAGCACCGACTTTCCT | 60 | 246 | E-8 | BC116039.1 |
|  |  |  |  |  |  |
| *IGF2*-P4 (F) | CAGCGAGCCTCCTGTCCA | 60 | 64 | E-7 | AY957981.1 |
| *IGF2*-P4 (R) | CACCAGCACCGACTTTCCT | 60 | 64 | E-8 | AY957981.1 |
|  |  |  |  |  |  |
| *IR* (F) | GGAGCCCAAGGAACCCAACG | 62 | 105 | E-13 | NC_007305.4 |
| *IR* (R) | AGAGCATAATGTCGGCGGGAGA | 62 | 105 | E-14 | NC_007305.4 |
|  |  |  |  |  |  |
| *IR*-A (F) | TCCTCAAGGAGCTGGAGGAGT | 59 | 89 | E-10 | AJ488553 |
| *IR*-A (R) | TTTCCTCGAAGGCCTGGGGAT | 59 | 89 | E-10,12 | AJ488553 |
|  |  |  |  |  |  |
| *IR*-B (F) | TCCTCAAGGAGCTGGAGGAGT | 59 | 110 | E-10 | AJ320235 |
| *IR*-B (R) | TAGCGTCCTCGGCAACAGG | 59 | 110 | E-11 | AJ320235 |
|  |  |  |  |  |  |
| *IGF1R* (F) | GATCCCGTGTTCTTCTACGTTC | 58 | 100 | E-13 | XM_606794.3 |
| *IGF1R* (R) | AAGCCTCCCACTATCAACAGAA | 58 | 100 | E-14 | XM_606794.3 |
|  |  |  |  |  |  |
| *IGF2R* (F) | GATGGTAATGAGCAGGCTTACC | 60 | 123 | E-47 | NM_174352.2 |
| *IGF2R* (R) | ATCTCCTCCATCAGCCACTC | 60 | 123 | E-48 | NM_174352.2 |

**S3 Table continued**

| **Target transcript** | **Primer sequence (5’ to 3’)** | **A.T. (°C)** | **F.L. (bp)** | **Exon (E)/intron (I)** | **Accession No.** |
| --- | --- | --- | --- | --- | --- |
| *IGFBP1* (F) | ACCAGCCCAGAGAATGTGTC | 59 | 119 | E-2 | X54979.1 |
| *IGFBP1* (R) | CTGATGGCATTCCAGAGGAT | 59 | 119 | E-2 | X54979.1 |
|  |  |  |  |  |  |
| *IGFBP2* (F) | CACATCCCCAACTGTGACAA | 58 | 114 | E-3 | NM_174555.1 |
| *IGFBP2* (R) | GATCAGCTTCCCGGTGTTAG | 58 | 114 | E-4 | NM_174555.1 |
|  |  |  |  |  |  |
| *IGFBP3* (F) | CTACGAGTCTCAGAGCACAG | 58 | 103 | E-2 | M76478.1 |
| *IGFBP3* (R) | GTGGTTCAGCGTGTCTTCC | 58 | 103 | E-3 | M76478.1 |
|  |  |  |  |  |  |
| *IGFBP4* (F) | ATGTGCCTGATGGAGAAAGG | 57 | 106 | E-4 | NM_174557.3 |
| *IGFBP4* (R) | GCCATCCTGTGACTTCCTGT | 57 | 106 | 5’ UTR | NM_174557.3 |
|  |  |  |  |  |  |
| *IGFBP5* (F) | CAAGCCAAGATCGAAAGAGACT | 60 | 85 | E-1 | NM_001105327.1 |
| *IGFBP5* (R) | AAGATCTTGGGCGAGTAGGTCT | 60 | 85 | E-2 | NM_001105327.1 |
|  |  |  |  |  |  |
| *IGFBP6* (F) | GGAGAGAATCCCAAGGAGAGTA | 60 | 100 | E-2 | NM_001040495.1 |
| *IGFBP6* (R) | GAGTGGTAGAGGTCCCCGAGT | 60 | 100 | E-2 | NM_001040495.1 |
|  |  |  |  |  |  |
| *IGFBP7* (F) | CTGCGAGGTCATCGGAATCCCCAC | 62 | 110 | E-2 | NW_001495197.4 |
| *IGFBP7* (R) | CCAGGTTGTCTCGGTCACCAGGCA | 62 | 110 | E-3 | NW_001495197.4 |
|  |  |  |  |  |  |
| *IGFBP8* (F) | GCTGACCTGGAGGAGAACATTA | 58 | 112 | E-4 | NT_182009.1 |
| *IGFBP8* (R) | CTCGGTATGTCTTCATGCTGG | 58 | 112 | E-5 | NT_182009.1 |
|  |  |  |  |  |  |
| *H19* (F) | TCAAGATGACAAGAGATGGTGCTA | 60 | 171 | E-3,4 | NR_003958.2 |
| *H19* (R) | GGTGTGGGTCGTCCGTTC | 60 | 171 | E-5 | NR_003958.2 |
|  |  |  |  |  |  |
| *AIRN* (F) | AATCTCTTGCGGAGTGTTCAT | 57 | 136 | I-2 (*IGF2R*) | DQ835615.1 |
| *AIRN* (R) | CTCTGTTGTATCGTGTCTTTCG | 57 | 136 | I-2 (*IGF2R*) | DQ835615.1 |
