# Supplementary material for: Atlas of tissue- and developmental stage specific gene expression for the bovine insulin-like growth factor (IGF) system
Source: PLoS One. 2018 Jul 12;13(7):e0200466. doi: 10.1371/journal.pone.0200466 (PMC6042742; doi:10.1371/journal.pone.0200466)
Supplement: S4 Table — (DOCX) [file pone.0200466.s004.docx]

**S4 Table.** **Details of forward (F) and reverse (R) primers used for amplification of transcripts of reference genes.** Primer sequences, annealing temperatures (AT), amplified fragment length (FL, in base pairs, bp), and GenBank accession number are shown.

| **Target gene** | **Primer sequence** | **AT (°C)** | **FL (bp)** | **Accession No.** |
| --- | --- | --- | --- | --- |
| *ACTB* (F) | CTCTTCCAGCCTTCCTTCCT | 62 | 245 | [NM_173979.3](http://www.ncbi.nlm.nih.gov/entrez/viewer.fcgi?db=nucleotide&id=75832053) |
| *ACTB* (R) | CCAATCCACACGGAGTACTTG | 62 | 245 | [NM_173979.3](http://www.ncbi.nlm.nih.gov/entrez/viewer.fcgi?db=nucleotide&id=75832053) |
|  |  |  |  |  |
| *RPS9* (F) | TAGGCGCAGACGGGCAAACA | 60 | 136 | [NM_001101152.2](http://www.ncbi.nlm.nih.gov/entrez/viewer.fcgi?db=nucleotide&id=402743974) |
| *RPS9* (R) | CCCATACTCGCCGATCAGCTTCA | 60 | 136 | [NM_001101152.2](http://www.ncbi.nlm.nih.gov/entrez/viewer.fcgi?db=nucleotide&id=402743974) |
|  |  |  |  |  |
| *UBB* (F) | AGATCCAGGATAAGGAAGGCAT | 62 | 198 | [NM_174133.2](http://www.ncbi.nlm.nih.gov/entrez/viewer.fcgi?db=nucleotide&id=31342776) |
| *UBB* (R) | GCTCCACCTCCAGGGTGAT | 62 | 198 | [NM_174133.2](http://www.ncbi.nlm.nih.gov/entrez/viewer.fcgi?db=nucleotide&id=31342776) |
|  |  |  |  |  |
| *H3F3A* (F) | ACTGCTACAAAAGCCGCTC | 60 | 231 | [XM_003586223.1](http://www.ncbi.nlm.nih.gov/entrez/viewer.fcgi?db=nucleotide&id=359066590) |
| *H3F3A* (R) | ACTTGCCTCCTGCAAAGCAC | 60 | 231 | [XM_003586223.1](http://www.ncbi.nlm.nih.gov/entrez/viewer.fcgi?db=nucleotide&id=359066590) |
|  |  |  |  |  |
| *TBP* (F) | GCAACAGTTCAGTAGTTATGAGCCAG | 60 | 164 | [NM_001075742.1](http://www.ncbi.nlm.nih.gov/entrez/viewer.fcgi?db=nucleotide&id=115496233) |
| *TBP* (R) | GAATAGGGTAGATGTTCTCAAAGGCT | 60 | 164 | [NM_001075742.1](http://www.ncbi.nlm.nih.gov/entrez/viewer.fcgi?db=nucleotide&id=115496233) |
|  |  |  |  |  |
| *VPS4A* (F) | GAAGACAGAAGGCTACTCGGGTG | 60 | 106 | [NM_001046615.1](http://www.ncbi.nlm.nih.gov/entrez/viewer.fcgi?db=nucleotide&id=114051384) |
| *VPS4A* (R) | ACAGACCTTTTTGAAGTGTGTTGCT | 60 | 106 | [NM_001046615.1](http://www.ncbi.nlm.nih.gov/entrez/viewer.fcgi?db=nucleotide&id=114051384) |
|  |  |  |  |  |
| *GAPDH* (F) | GGGTCATCATCTCTGCACCT | 62 | 173 | [NM_001034034.2](http://www.ncbi.nlm.nih.gov/entrez/viewer.fcgi?db=nucleotide&id=402744670) |
| *GAPDH* (R) | CATAAGTCCCTCCACGATGC | 62 | 173 | [NM_001034034.2](http://www.ncbi.nlm.nih.gov/entrez/viewer.fcgi?db=nucleotide&id=402744670) |
