# Supplementary material for: Atlas of tissue- and developmental stage specific gene expression for the bovine insulin-like growth factor (IGF) system
Source: PLoS One. 2018 Jul 12;13(7):e0200466. doi: 10.1371/journal.pone.0200466 (PMC6042742; doi:10.1371/journal.pone.0200466)
Supplement: S5 Table — (DOCX) [file pone.0200466.s005.docx]

**S5 Table.** **Stability values for studied reference genes and best combination of genes for different tissues as calculated by NormFinder.**

|  | **Placenta** | **Heart** | **Kidney** | **Liver** | **Lung** | **Muscle** |
| --- | --- | --- | --- | --- | --- | --- |
| ***ACTB*** | 0.209 | 0.264 | 0.323 | 0.352 | 0.145 | 0.302 |
| ***RPS9*** | 0.209 | 0.236 | 0.312 | 0.387 | 0.213 | 0.260 |
| ***UBB*** | 0.307 | 0.305 | 0.374 | 0.304 | 0.294 | 0.543 |
| ***H3F3A*** | _ | 0.194 | 0.254 | 0.381 | 0.212 | 0.328 |
| ***TBP*** | 0.212 | 0.252 | 0.296 | 0.393 | 0.189 | 0.330 |
| ***VPS4A*** | 0.123 | 0.144 | 0.259 | 0.242 | 0.168 | 0.273 |
| ***GAPDH*** | 0.183 | _ | _ | _ | _ | _ |
| ***H3F3A* / *VPS4A*** | _ | _ | 0.180 | _ | _ | _ |
| ***GAPDH* / *VPS4A*** | 0.132 | _ | _ | _ | _ | _ |
| ***ACTB* / *VPS4A*** | _ | 0.132 | _ | _ | _ | _ |
| ***ACTB* / *RPS9*** | _ | _ | _ | _ | 0.124 | _ |
| ***ACTB* / *H3F3A*** | _ | _ | _ | _ | _ | 0.205 |
