# Supplementary material for: Atlas of tissue- and developmental stage specific gene expression for the bovine insulin-like growth factor (IGF) system
Source: PLoS One. 2018 Jul 12;13(7):e0200466. doi: 10.1371/journal.pone.0200466 (PMC6042742; doi:10.1371/journal.pone.0200466)
Supplement: S6 Table — (DOCX) [file pone.0200466.s006.docx]

**S6 Table.** **Comparison of changes in gene expression in the bovine IGF system between Day 48 embryo and Day 153 fetal stages.** Fold change from embryo to fetal stage was calculated as transcript abundance for a given gene in fetal tissue (FT) divided by the transcript abundance for same gene in embryonic tissue (ET). Geometric means of fold changes calculated for each gene across tissues (‘mean’) and geometric means of fold changes for studied gene groups, i.e., ligands (*IGF1*, *IGF2*), receptors (*IGF1R*, *IGF2R*, *IR*), binding proteins (*IGFBP1 - 8*) and long non-coding RNAs (*H19*, *AIRN*) across tissues (‘group mean’), are also shown.

|  |  | **Brain**  **(FT/ET)** | **Heart**  **(FT/ET)** | **Liver**  **(FT/ET)** | **Mean**  **(FT/ET)** | **Group mean (FT/ET)** |
| --- | --- | --- | --- | --- | --- | --- |
|  |  |  |  |  |  |  |
| **Ligands** | *IGF1* | 6.4 :1 | 2.5 :1 | 1.2 :1 | 2.6 :1 | 1.9 :1 |
|  | *IGF2* | 1.3 :1 | 2 :1 | 1.1 :1 | 1.4 :1 |  |
|  |  |  |  |  |  |  |
| **Receptors** | *IGF1R* | 1 :1.8 | 2.9 :1 | 1 :2.3 | 1 :1.1 | 1.7 :1 |
|  | *IGF2R* | 4.1 :1 | 4.9 :1 | 2.5 :1 | 3.7 :1 |  |
|  | *IR* | 1 :1 | 5.8 :1 | 1 :1.5 | 1.6 :1 |  |
|  |  |  |  |  |  |  |
| **Binding proteins** | *IGFBP1* | 1 :1.1 | 1 :1.6 | 1.2 :1 | 1 :1.2 | 2 :1 |
|  | *IGFBP2* | 1 :14 | 1.1 :1 | 4.8 :1 | 1 :1.4 |  |
|  | *IGFBP3* | 2.3 :1 | 1.8 :1 | 1.5 :1 | 1.8 :1 |  |
|  | *IGFBP4* | 1.4 :1 | 2.7 :1 | 1.1 :1 | 1.6 :1 |  |
|  | *IGFBP5* | 3.6 :1 | 7.9 :1 | 1.6 :1 | 3.6 :1 |  |
|  | *IGFBP6* | 5.7 :1 | 3.3 :1 | 2.1 :1 | 3.4 :1 |  |
|  | *IGFBP7* | 4.4 :1 | 3.6 :1 | 5.1 :1 | 4.3 :1 |  |
|  | *IGFBP8* | 2.4 :1 | 2.2 :1 | 2.4 :1 | 2.3 :1 |  |
|  |  |  |  |  |  |  |
| **lncRNAs** | *AIRN* | 3.5 :1 | 6.6 :1 | 1.7 :1 | 3.4 :1 | 1.4 :1 |
|  | *H19* | 1 :1.7 | 1 :2 | 1 :1.5 | 1 :1.7 |  |
|  |  |  |  |  |  |  |
|  | Mean | 1.6 :1 | 2.5 :1 | 1.5 :1 |  |  |
