# Supplementary material for: Atlas of tissue- and developmental stage specific gene expression for the bovine insulin-like growth factor (IGF) system
Source: PLoS One. 2018 Jul 12;13(7):e0200466. doi: 10.1371/journal.pone.0200466 (PMC6042742; doi:10.1371/journal.pone.0200466)
Supplement: S7 Table — (DOCX) [file pone.0200466.s007.docx]

**S7 Table.** **Comparison of changes in gene expression in the bovine IGF system between Day 153 fetal and 12-14 month juvenile stages.** Fold change from fetal to juvenile stage was calculated as transcript abundance for a given gene in fetal tissue (FT) divided by the transcript abundance for same gene in juvenile tissue (JT). Geometric means of fold changes calculated for each gene across studied tissues (‘mean’) and geometric means of fold changes for studied gene groups, i.e., ligands (*IGF1*, *IGF2*), receptors (*IGF1R*, *IGF2R*, *IR*), binding proteins (*IGFBP1 - 8*) and long non-coding RNAs (*H19*, *AIRN*) across tissues (‘group mean’), are also shown.

|  |  | **Brain**  **(FT/JT)** | **Heart**  **(FT/JT)** | **Liver**  **(FT/JT)** | **Kidney**  **(FT/JT)** | **Lung**  **(FT/JT)** | **Muscle**  **(FT/JT)** | **Mean**  **(FT/JT)** | **Group mean (FT/JT)** |
| --- | --- | --- | --- | --- | --- | --- | --- | --- | --- |
|  |  |  |  |  |  |  |  |  |  |
| **Ligands** | *IGF1* | 8.8 :1 | 3.6 :1 | 1 :36.5 | 8.6 :1 | 60.7 :1 | 10.6 :1 | 4.1 :1 | 12.1 :1 |
|  | *IGF2* | 6.1 :1 | 31 :1 | 4 :1 | 122.2 :1 | 355.7 :1 | 60.1 :1 | 35.4: 1 |  |
|  |  |  |  |  |  |  |  |  |  |
| **Receptors** | *IGF1R* | 6.4 :1 | 3.6 :1 | 3.8 :1 | 20.2 :1 | 17.3 :1 | 2.6 :1 | 6.6 :1 | 5.7 :1 |
|  | *IGF2R* | 5.8 :1 | 36.3 :1 | 6.1 :1 | 25.6 :1 | 58.6 :1 | 2.4 :1 | 13 :1 |  |
|  | *IR* | 1.9 :1 | 2.1 :1 | 1.6 :1 | 3.7 :1 | 2.8 :1 | 1.5 :1 | 2.1 :1 |  |
|  |  |  |  |  |  |  |  |  |  |
| **Binding proteins** | *IGFBP1* | 4.9 :0 | 6 :1 | 2.3 :1 | 4.2 :1 | 0 :0 | 4.1 :1 | 4.1 :1 | 4.3 :1 |
|  | *IGFBP2* | 2.6 :1 | 67.2 :1 | 3.3 :1 | 16.4 :1 | 1.4 :1 | 105.8 :1 | 10.6 :1 |  |
|  | *IGFBP3* | 3.8 :1 | 4.1 :1 | 1.3 :1 | 5.9 :1 | 14.7 :1 | 2.1 :1 | 4 :1 |  |
|  | *IGFBP4* | 8.6 :1 | 11.2 :1 | 1 :4.5 | 5.8 :1 | 6.6 :1 | 18.3 :1 | 5 :1 |  |
|  | *IGFBP5* | 16.4 :1 | 2.7 :1 | 4.4 :1 | 5.9 :1 | 13 :1 | 2.5 :1 | 5.7 :1 |  |
|  | *IGFBP6* | 1.5 :1 | 1.4 :1 | 1.1 :1 | 6.1 :1 | 6 :1 | 1.4 :1 | 2.2 :1 |  |
|  | *IGFBP7* | 3.3 :1 | 1.9 :1 | 1.3 :1 | 9.9 :1 | 2.2 :1 | 12.7 :1 | 3.6 :1 |  |
|  | *IGFBP8* | 3 :1 | 2.6 :1 | 5.6 :1 | 6.7 :1 | 2.1 :1 | 1.8 :1 | 3.2 :1 |  |
|  |  |  |  |  |  |  |  |  |  |
| **lncRNAs** | *AIRN* | 2.6 :1 | 7.5 :1 | 9.8 :1 | 58.5 :1 | 29 :1 | 3.3 :1 | 10.1 :1 | 16 :1 |
|  | *H19* | 2.9 :0 | 65.7 :1 | 36.8 :1 | 101.2 :1 | 41.5 :1 | 8.9 :1 | 25.3 :1 |  |
|  |  |  |  |  |  |  |  |  |  |
|  | Mean | 4.3 :1 | 7 :1 | 2.2 :1 | 13.2 :1 | 12.9 :1 | 5.7 :1 |  |  |
