# Supplementary material for: Atlas of tissue- and developmental stage specific gene expression for the bovine insulin-like growth factor (IGF) system
Source: PLoS One. 2018 Jul 12;13(7):e0200466. doi: 10.1371/journal.pone.0200466 (PMC6042742; doi:10.1371/journal.pone.0200466)
Supplement: S8 Table — (DOCX) [file pone.0200466.s008.docx]

**S8 Table.** **Comparison of changes in gene expression in the bovine IGF system of placenta from embryo stage to term.** Fold change from embryo to fetal stage and from fetal to term (C-Section) stage was calculated as transcript abundance for a given gene in embryonic placenta (EP) divided by transcript abundance for same gene in fetal placenta (FP) and as transcript abundance for a given gene in fetal placenta (FP) divided by transcript abundance for same gene at term (TP), respectively. Geometric means of fold changes for studied gene groups, i.e., ligands (*IGF1*, *IGF2*), receptors (*IGF1R*, *IGF2R*, *IR*), binding proteins (*IGFBP1 - 8*) and long non-coding RNAs (*H19*, *AIRN*) across tissues (‘group mean’), are also shown.

|  |  | **Placenta**  **(EP/FP)** | **Group mean (EP/FP)** |  | **Placenta**  **(FP/TP)** | **Group mean (FP/TP)** |
| --- | --- | --- | --- | --- | --- | --- |
|  |  |  |  |  |  |  |
| **Ligands** | *IGF1* | 7.2 :1 | 2.9 :1 |  | 2.5 :1 | 2.5 :1 |
|  | *IGF2* | 1.2 :1 |  |  | 2.6 :1 |  |
|  |  |  |  |  |  |  |
| **Receptors** | *IGF1R* | 2.4 :1 | 1.1 :1 |  | 1 :3.9 | 1.1 :1 |
|  | *IGF2R* | 1 :1.7 |  |  | 6.2 :1 |  |
|  | *IR* | 1 :1 |  |  | 1 :1.1 |  |
|  |  |  |  |  |  |  |
| **Binding proteins** | *IGFBP1* | 0 :23.9 | 1 :1 |  | 3.4 :1 | 1.4 :1 |
|  | *IGFBP2* | 1 :1 |  |  | 9.6 :1 |  |
|  | *IGFBP3* | 3.2 :1 |  |  | 1 :2.5 |  |
|  | *IGFBP4* | 1.7 :1 |  |  | 4.3 :1 |  |
|  | *IGFBP5* | 1 :1.5 |  |  | 1.1 :1 |  |
|  | *IGFBP6* | 1.5 :1 |  |  | 1.2 :1 |  |
|  | *IGFBP7* | 3.8 :1 |  |  | 1 :2.9 |  |
|  | *IGFBP8* | 1 :1.3 |  |  | 1 :1.7 |  |
|  |  |  |  |  |  |  |
| **lncRNAs** | *AIRN* | 1.2 :1 | 1.5 :1 |  | 6.2 :1 | 2.9 :1 |
|  | *H19* | 1.9 :1 |  |  | 1.4 :1 |  |
|  |  |  |  |  |  |  |
|  | Mean | 1.2 :1 |  |  | 1.6 :1 |  |
