# Supplementary material for: Atlas of tissue- and developmental stage specific gene expression for the bovine insulin-like growth factor (IGF) system
Source: PLoS One. 2018 Jul 12;13(7):e0200466. doi: 10.1371/journal.pone.0200466 (PMC6042742; doi:10.1371/journal.pone.0200466)
Supplement: S9 Table — (DOCX) [file pone.0200466.s009.docx]

**S9 Table. Relative contribution of *IGF2* promoter-specific transcripts to global *IGF2* transcript abundance with 95% confidence intervals (lower and upper endpoints) for studied fetal tissues.**

| **Transcript** | **Tissue** | **Estimated contribution to global *IGF2* (%)^a^** | **Lower endpoint (%)** | **Upper endpoint (%)** |
| --- | --- | --- | --- | --- |
| ***IGF2*-P2e5** | **Muscle** | 4 | 1.8 | 8.4 |
| ***IGF2*-P3** | **Muscle** | 3 | 0.6 | 8.8 |
| ***IGF2*-P4** | **Muscle** | 28 | 20 | 37.2 |
| ***IGF2*-P0** | **Muscle** | 30 | 22.3 | 35.6 |
| ***IGF2*-P2e4** | **Muscle** | 35 | 25.3 | 43.3 |
| ***IGF2*-P2e5** | **Liver** | 1 | 0.2 | 4.7 |
| ***IGF2*-P3** | **Liver** | 1 | 0.6 | 3.8 |
| ***IGF2*-P4** | **Liver** | 53 | 46.8 | 58.7 |
| ***IGF2*-P2e4** | **Liver** | 44 | 37.1 | 49.3 |
| ***IGF2*-P2e5** | **Heart** | 24 | 10.2 | 40.3 |
| ***IGF2*-P3** | **Heart** | 4 | 1.3 | 16.4 |
| ***IGF2*-P4** | **Heart** | 72 | 49.4 | 86.0 |
| ***IGF2*-P2e5** | **Lung** | 31 | 16.7 | 41.7 |
| ***IGF2*-P3** | **Lung** | 8 | 3.9 | 16.6 |
| ***IGF2*-P4** | **Lung** | 61 | 43.3 | 78.7 |
| ***IGF2*-P2e5** | **Placenta** | 24 | 5 | 31.8 |
| ***IGF2*-P3** | **Placenta** | 12 | 1.7 | 31.6 |
| ***IGF2*-P4** | **Placenta** | 64 | 41.5 | 85.5 |
| ***IGF2*-P2e5** | **Kidney** | 3 | 0.3 | 17.1 |
| ***IGF2*-P3** | **Kidney** | 7 | 2.3 | 24.5 |
| ***IGF2*-P4** | **Kidney** | 90 | 67.2 | 95.5 |

^a^ The relative contribution of each promoter-specific transcript to global *IGF2* transcript abundance was calculated by Johnson’s Relative Weight procedure.
